# Phylogenetic Distinctiveness of Middle Eastern and Southeast Asian Village Dog Y Chromosomes Illuminates Dog Origins

**DOI:** 10.1371/journal.pone.0028496

**Published:** 2011-12-14

**Authors:** Sarah K. Brown, Niels C. Pedersen, Sardar Jafarishorijeh, Danika L. Bannasch, Kristen D. Ahrens, Jui-Te Wu, Michaella Okon, Benjamin N. Sacks

**Affiliations:** 1 Canid Diversity and Conservation Laboratory, Center for Veterinary Genetics, University of California Davis, Davis, California, United States of America; 2 Veterinary Genetics Laboratory, University of California at Davis, Davis, California, United States of America; 3 Center for Companion Animal Health, School of Veterinary Medicine, University of California Davis, Davis, California, United States of America; 4 Department of Medicine and Epidemiology, School of Veterinary Medicine, University of California Davis, Davis, California, United States of America; 5 Department of Clinical Sciences, School of Veterinary Medicine, Shiraz University, Shiraz, Iran; 6 Department of Population, Health and Reproduction, School of Veterinary Medicine, University of California Davis, Davis, California, United States of America; 7 Biological Sciences Department, California State University Sacramento, Sacramento, California, United States of America; 8 Department of Veterinary Medicine, National Chiayi University, Chiayi City, Taiwan, Republic of China; 9 Ruah Midbar Kennel for Desert Bred Salukis, Herzliya, Israel; University of Western Ontario, Canada

## Abstract

Modern genetic samples are commonly used to trace dog origins, which entails untested assumptions that village dogs reflect indigenous ancestry or that breed origins can be reliably traced to particular regions. We used high-resolution Y chromosome markers (SNP and STR) and mitochondrial DNA to analyze 495 village dogs/dingoes from the Middle East and Southeast Asia, along with 138 dogs from >35 modern breeds to 1) assess genetic divergence between Middle Eastern and Southeast Asian village dogs and their phylogenetic affinities to Australian dingoes and gray wolves (*Canis lupus*) and 2) compare the genetic affinities of modern breeds to regional indigenous village dog populations. The Y chromosome markers indicated that village dogs in the two regions corresponded to reciprocally monophyletic clades, reflecting several to many thousand years divergence, predating the Neolithic ages, and indicating long-indigenous roots to those regions. As expected, breeds of the Middle East and East Asia clustered within the respective regional village dog clade. Australian dingoes also clustered in the Southeast Asian clade. However, the European and American breeds clustered almost entirely within the Southeast Asian clade, even sharing many haplotypes, suggesting a substantial and recent influence of East Asian dogs in the creation of European breeds. Comparison to 818 published breed dog Y STR haplotypes confirmed this conclusion and indicated that some African breeds reflect another distinct patrilineal origin. The lower-resolution mtDNA marker consistently supported Y-chromosome results. Both marker types confirmed previous findings of higher genetic diversity in dogs from Southeast Asia than the Middle East. Our findings demonstrate the importance of village dogs as windows into the past and provide a reference against which ancient DNA can be used to further elucidate origins and spread of the domestic dog.

## Introduction

Archaeology and DNA studies indicate that dogs evolved from or share a recent common ancestor with the gray wolf (*Canis lupus*) 12,000–40,000 years BP, and that they spread rapidly throughout Eurasia and the Americas at the end of the last ice age [1]–[6]. However, controversy persists over where dogs originated, with most evidence cited in favor of Europe [7]–[9], the Middle East [10]–[12], or Southeast Asia [6], [13], [14]. One problem potentially confounding this question is uncertainty in the links between extant dogs and the original canine inhabitants of those same regions. Modern DNA studies implicitly assume that today's dogs reflect the deeper ancestry of their putative home regions, which may not be the case.

In particular, the genomes of modern dog breeds reflect geographically diverse sources, owing to relatively recent and extensive, post-Victorian efforts to create a diversity of specialized phenotypes [4], [15], [16]. Stray dogs of present Europe and North America primarily reflect secondary admixtures of these same recently created breeds and therefore can be expected to equally misrepresent the ancestry indigenous to those regions [17]. In contrast, village dogs have occurred throughout Asia and Africa continuously, more or less independently of modern breeds, and therefore are more likely to reflect the deeper indigenous ancestry of their regions [17]–[20]. The fundamental aim of our study was to test this hypothesis, in particular regarding village dogs from Southeast Asia and the Middle East, two of the leading candidate regions hypothesized to have hosted dog origins [6], [10]–[14]. Identification of indigenous village dog populations is an essential step in tracing the ultimate origins of the dog, and also can help elucidate proximate geographic origins of modern breeds [18]–[21], which in turn have been used extensively in DNA studies aimed at elucidating dog origins [11], [12], [14].

In principle, comparison to ancient DNA would be the most straightforward means of testing the indigenousness of extant dogs. However, the small number of ancient samples typically available and resolution of the DNA most accessible in those samples (mitochondrial) limit the practical utility of this approach. An alternative approach is to assess the genetic divergence between relatively large samples of extant village dogs from multiple regions to infer population ages. Specifically, if populations reflect primarily indigenous ancestry, their genetic divergence should reflect thousands of years' isolation, whereas if they are heavily admixed with modern Western breeds, they should reflect little genetic divergence. Mitochondrial DNA (mtDNA) sequences are commonly used in phylogeographic studies of plants and animals, including dogs [3], [4], [6], [14], because their mutational history exposes their genealogy. However, these molecules mutate too slowly (even the entire mtDNA genome) to enable precise estimates of divergence on the timescale of recent dog evolution. For the D-loop fragment typically used in dog studies, <10% of haplotypes are expected to have accumulated mutations in the past 10,000 years [3], [6], [14]. The mitochondrion also represents only a single outcome of the genealogical history. Therefore, a second, independent clonally inherited marker with a higher mutation rate could potentially clarify much of the existing ambiguity, particularly if examined in indigenous village dogs. The Y chromosome provides such a marker.

DNA markers on the non-recombining region of the Y chromosome (NRY) have been used to great advantage in studies of several domestic species and humans [22]–[24], but their use for dogs has been restricted largely to breeds, for example, confirming the very recent (<400 years) origins of most modern breeds [21], [25], [26]. Y chromosome markers have never been studied in village dogs, which, if indigenous, are essential for determining the more ancient origins of domestication. Moreover, data from highly conserved NRY SNPs can be combined with data from rapidly mutating single-tandem repeat (STR or microsatellite) markers on the NRY to provide resolution over a broad window of time, e.g., covering 10^2^–10^4^ generations.

Our first objective was to determine whether village dogs from the Middle East and Continental and Island Southeast Asia were indigenous to those regions or, alternatively, secondary products of a post-Victorian expansion of Western breed dogs. The second objective was to determine whether modern breeds could be traced to either of these putative indigenous village dog populations. We used highly resolved Y-chromosome SNP-STR haplotypes to assess the approximate minimum age and genetic similarity of these village dog populations. To address uncertainty in STR mutation rates, estimates of divergence time were calibrated using Australian dingoes and Bali dogs, both of which are known to have been isolated for several thousand years based on independent evidence [17]–[19], [27], [28]. We then compared NRY haplotypes of these village dogs to those of 124 dogs representing >35 contemporary breeds to assess phylogenetic affinities with the two “geo-referenced” village dog populations. Lastly, we compared the Y-STR portion of these haplotypes to 818 previously published breed dog Y-STR haplotypes to better assess the generality of our findings. We also analyzed mtDNA in village dogs to further test previous findings of higher diversity in Southeast Asia than the Middle East, but with a purely village-dog sample, including a larger number than previously examined from the Middle East. Because we sampled different areas of Southeast Asia and the Middle East than the previous studies [6], [14], comparison with these data allowed us to assess the spatial extent of these regional populations and, therefore, to assess robustness of conclusions to particularities of sampling locations.

## Materials and Methods

### Ethics Statement

All procedures involving animals were reviewed and approved by the University of California, Davis, Animal Care and Use Committee (Protocol No. 16643).

### Samples

We sampled 9 wild canids and 633 dogs for this study, including 480 village dogs (300 males) from the Middle East and Southeast Asia, 15 Australian dingoes (5 males), 45 desert-bred Salukis (31 males), and 93 male breed dogs from 35 additional breeds or mixtures of breeds. Blood, tissue, or buccal swabs were obtained from the wild canids, including gray wolves from Iran (n = 3), China (n = 1), and the Yukon, Canada (n = 3), along with a black-backed jackal (*Canis mesomelas*) and a dhole (*Cuon alpinus*) from captivity. Buccal swabs were collected from dogs. Most village dogs (mainland and Island Southeast Asia) were captured in the course of spay-neuter programs (Figure S1). Middle Eastern village dogs were sampled from Iran (Shiraz, n = 180; Kerman, n = 31; Kazerun, n = 22) along with desert-bred Salukis from Israel (n = 45). Roughly a quarter of the Southeast Asian village dogs were from the mainland (i.e., Thailand, n = 57), directly south of where Pang et al. [14] hypothesized dogs were domesticated. The remainder were from Islands in Southeast Asia: Taiwan (n = 40), Brunei (n = 27), Bali (n = 97), and the Philippines (n = 26), along with 15 dingoes from Fraser Island, Australia, where introgression from domestic dogs was expected to be minimal [29]. The assumption that dingoes were indigenous was also verified based on mtDNA in reference to published dingo haplotypes [28].

Our Southeast Asian sample included dogs from relatively large island populations that were geographically and historically linked to mainland Southeast Asia and, therefore, reflected mainland-Southeast Asian ancestry [17], [18], [27]. To assess whether the founding histories of Island populations substantially reduced genetic diversity of our total Southeast Asian sample, we compared mtDNA haplotype diversity to that of the mainland Southeast Asian sample of Pang et al. [14]. We also took advantage of independently timed founding histories for dogs of Bali and Australia (i.e., dingoes) to estimate evolutionary mutation rates (slower than pedigree-based mutation rates; more accurate for divergence estimates [22]) and calibrate temporal estimates [17], [28].

### Laboratory methods

DNA was extracted from buccal swabs using a standard protocol [30] and from tissue and blood samples using the Qiagen DNeasy kit, according to manufacturer's instructions. A 402 bp portion of the mtDNA hypervariable region I (D-loop) was then sequenced using the following primers: CCCTGACACCCCTACATTCA (forward) and CTTATATGCATGGGGCAAACC (reverse) and Big Dye sequencing chemistry (Applied Biosystems, Inc.). Males were genotyped using 5 dinucleotide-repeat STRs from the NRY, including 650−79.2, 650−79.3, 990−35 [21], MS34A, and MS41B [31] in two separate multiplex reactions as previously described [21], [31]. The thermal profile for both PCR reactions was 1 min at 95 C°, followed by 30 cycles of 30 s at 95 C°, 30 s at 56 C°, 1 min at 72 C°, and a final extension at 72 C° for 30 min. An ABI 3730 capillary sequencer was used for electrophoresis (Applied Biosystems, Inc.) and alleles were scored using STRand [32].

Male samples were genotyped at 11 NRY SNP loci [25] using iPLEX Sequenom MassARRAY system (Sequenom Inc., San Diego, CA) and our own PCR (Table S1) and extension (Table S2) primers. We were unable to develop usable primers for 3 additional published NRY SNPs that would have separated haplotypes 1–4 [25], so these published haplotypes were merged as a single haplotype in the present study.

### Data analyses

Mitochondrial DNA sequences were used both as a means of assessing the similarity of our samples to the nearby samples of Pang et al. [14] from Southeast Asia and the Middle East (i.e., “Southwest Asia”) and to reevaluate their findings based on a larger sample of village dogs from the Middle East. We compared samples in terms of haplotype diversity as well as the proportion of “universally occurring haplotypes” (UT), which is expected to be lowest in ancestral populations and highest in derived ones [14].

We constructed phylogenetic networks based on Y-SNPs, Y-STRs, and the combination of both markers. The Y-SNP networks were used to coarsely characterize the deeper phylogenetic structure in village dogs, whereas the Y STR networks provided far greater resolution with respect to recent divergence in the same village dogs and enabled us to directly compare 818 previously published breed dog haplotypes [21]. However, to more accurately estimate the topology, especially branch lengths, it was desirable to construct networks using both markers.

The Y chromosome networks were constructed using program Network 4.50 [33] with default settings, *r* = 2 and є = 0. We first constructed median-joining networks [34] and then applied a reduced-median analysis to create final networks, which was previously shown to optimize phylogenetic accuracy based on Y-chromosome STRs and SNPs [22]. The STR loci were weighted as per Bannasch et al. [21], inversely to their variance. Given the much lower rate of nuclear substitutions relative to STR mutations, SNP loci were each weighted as the maximum allowed by the program, which was 10 times the highest STR weight. Specifically, STR loci were weighted as follows: 650−79.2 = 5, 650−79.3 = 2, 990−35 = 9, MS34A = 6, MS41B = 1, and SNPs were weighted 90. In contrast to bifurcating trees, for which bootstrapping is typically used as a post-hoc measure of confidence, network approaches integrate statistical parsimony criteria into the network construction algorithm, such that internal nodes connected by a single line imply statistical support (i.e., 95% parsimony; [33]–[35]).

### Estimating divergence time

In principle, use of coalescent models and Markov chain Monte Carlo (MCMC) approaches can be used to estimate population (or clade) splitting times [36]. However, numerous iterations of this approach with Y chromosome data in program Batwing using different sets of reasonable priors and demographic models indicated results were too sensitive to priors (especially STR mutation rate) and too imprecise to provide an informative analysis of the existing data set. Therefore, a more straightforward approach based directly on the network-reconstructions was employed. We calculated the average number of mutations separating ancestral nodes from all descendent nodes and values (i.e., ρ) by a range of mutation rates to estimate the mutation-scaled age of clades [22], [37]. This approach depends primarily on the accumulation of mutations and is reasonably robust to population structure, but tends to exhibit a biased variance when populations have undergone long-term bottlenecks [38]. The biased variance results in estimated “95%” confidence intervals (under the normal approximation) that tend, in fact, to contain the true value only 65% of the time. However, type I errors tend to take the form of time underestimates, making this a conservative approach for this study [38]. A more important source of uncertainty was the mutation rate of the markers, including the per-generation rate and the average generation time, both of which were unknown. Therefore, we estimated generation-independent mutation rates by calibrating to the Bali village dog population, which was founded and subsequently isolated from other Southeast Asian populations ∼3,000 BP [18], [27]. Specifically, we estimated the average ρ (age in units of accumulated mutations) of endemic Bali clades and divided by 3,000 years to produce an estimate of the yearly haplotype mutation rate.

## Results

### mtDNA diversity in village dogs

Village dogs from the Middle East (*n* = 200) and Southeast Asia (*n* = 231) were sequenced at the mtDNA locus. This analysis revealed a total of 54 HVI haplotypes, of which 17 were novel (Genbank Accession Nos. HQ287728–HQ287744). The 15 Australian dingoes from Fraser Island all had indigenous dingo haplotypes (din3, din15, din20; [28]), confirming their indigenous status [29]. Despite differences in sample composition and specific locations between our study and that of Pang et al. [14], estimates of gene diversity were nearly identical between studies for both the Middle East (0.87, *n* = 199 vs. 0.86, *n* = 37, respectively) and Southeast Asia (0.92, *n* = 253 vs. 0.94, *n* = 612, respectively). The 402-bp equivalents of all of the widespread ancestral haplotypes previously identified as universally occurring haplotypes (UTs) were found in at least one of the two populations (Table 1; Table S3). In total, UTs made up 47% and 85% of the Southeast Asian and Middle Eastern village dog samples, respectively, similar to previous findings in nearby regions [14]. None of the haplotypes in subclades previously found only in Southeast Asia (a2–a5, b2; [14]) were found in village dogs from the Middle East in the present study despite our considerably larger samples size. Additionally, three times as many new (i.e., previously undescribed) haplotypes were found in Southeast Asia (*n* = 12) than the Middle East (*n* = 4) (Table S3, S4), even though Southeast Asia had been more extensively sampled (*n* = 612 village dogs) than the Middle East (*n* = 37 village dogs) in the past [14]. Two (17%) of the novel Southeast Asian haplotypes were from the mainland of Southeast Asia (Thailand). Diversity varied considerably among our sampling locations within regions but the 4 locations with the highest diversity were in Southeast Asia, including Thailand, Taiwan, Philippines, and Brunei (Table S5). Thus, the present mtDNA results confirmed previous findings of higher mtDNA diversity in Southeast Asia than the Middle East, and did so based solely on village dogs.

**Table 1:** Distribution of mtDNA haplotypes (402-bp from hypervariable region I) corresponding to “universally occurring” types (UT; [14]) in 431 village dogs from Southeast Asia and the Middle East.

UT haplotype	Southeast Asia	Middle East
A2	3	–
A11	19	43
A16, A17^a^	18	11
A18,A20^a^	19	16
A19	9	44
A22	–	3
A3	5	3
B1	18	27
B6	5	11
C1	9	7
C3	3	4
C5	1	–
Total No. UT	109	169
Total No. non-UT	131	75

a Haplotype pairs, A16/A17 and A18/A20, were indistinguishable from the 402-bp region examined in our study.

It is noteworthy that village dogs from Bali (n = 94) exhibited a lower haplotype diversity than dogs from other islands in Southeast Asia or sampling sites in the Middle East (Table S5). This was consistent with a long-term isolation of the Bali dog population [18]. Additionally, 5 haplotypes (of 18 total) were found only on Bali. One of these novel haplotypes (V9) differed by 2 substitutions from the nearest widespread haplotype (A11) and the other 4 differed by a single substitution from the A11 (n = 1) or A116 (n = 3; Table S4). Assuming that all novel haplotypes on Bali were endemic and derived in-situ, the average number of mutations accumulating since the population's founding was 0.0957 in the 94 village dogs. This figure can be divided by 402 bp times the previously estimated site-specific mutation rate of 7.2 * 10^−8^ per year [14] to produce a corresponding estimate of 3,300 years isolation, which agrees with archaeological evidence [18], [27].

### Y chromosome phylogeny of village dogs, dingoes, and wolves

**Figure 1:**
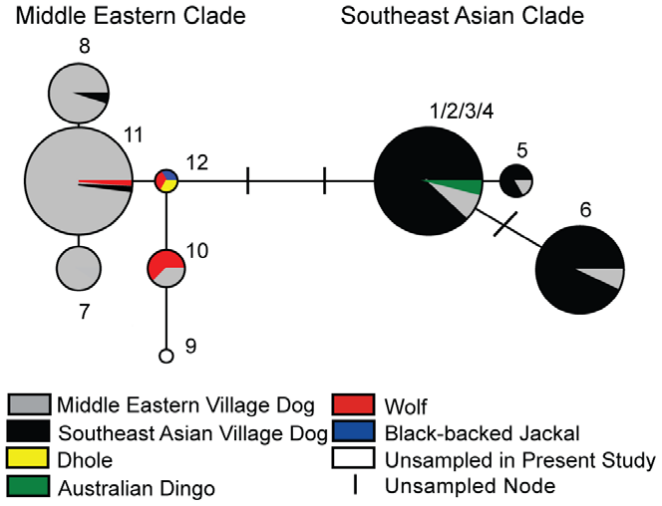
Y chromosome SNP haplotype network of village dogs and wild canids. Samples include Southeast Asian village dogs (*n* = 159), Australian dingoes (*n* = 5), Middle Eastern village dogs (*n* = 136), wolves (*n* = 7), dhole (*n* = 1) and black-backed jackal (*n* = 1), which form a primarily Middle East village dog clade, including wolf and outgroup taxa, and a primarily Southeast Asian village dog clade. The 8 haplotypes were based on 11 SNP mutations and included 6 found previously (haplotypes 1–9, [25]) and 3 new ones (haplotypes 10–12; reflected previously as unsampled nodes). Haplotypes 1–4 could not be distinguished based on the 11 sites genotyped in our study. Size of circle is proportional to sample size. Haplotypes of 48 village dogs were incomplete and imputed based on 8–10 SNPs and associations with similar STR haplotypes (Table S7).

The more ancient phylogenetic relationships of the Southeast Asian and Middle Eastern village dog patrilines were revealed by the NRY SNPs in 300 village dogs and dingoes, along with 7 gray wolves, and an out-group of 1 dhole and 1 black backed jackal. The 8 resulting haplotypes (Table S6) fell into two distinct clades, which corresponded to Southeast Asian and Middle Eastern village dogs (Figure 1; Table S7). All 5 dingoes bore the same haplotype found in most Southeast Asian village dogs, corresponding to the interior node of the Southeast Asian clade (Figure 1). The interior-most node of the Middle Eastern clade corresponded to the new haplotype 12, which was previously unsampled [25]. This novel haplotype was shared by the dhole, black-backed jackal, and a wolf from China, indicating that it was the most ancestral node (i.e., the root) in the network. The haplotypes of the other wolves also clustered closer to the Middle Eastern clade, including three wolves from Canada and two from Iran sharing haplotype 10 and one from Iran with haplotype 11. The Southeast Asian clade was more distantly derived (3 substitutions) from this ancestral haplotype, suggesting it reflected a more ancient dog clade, a distinct wolf patriline not sampled in this study, or distortion due to ascertainment biases associated with SNP discovery.

**Figure 2:**
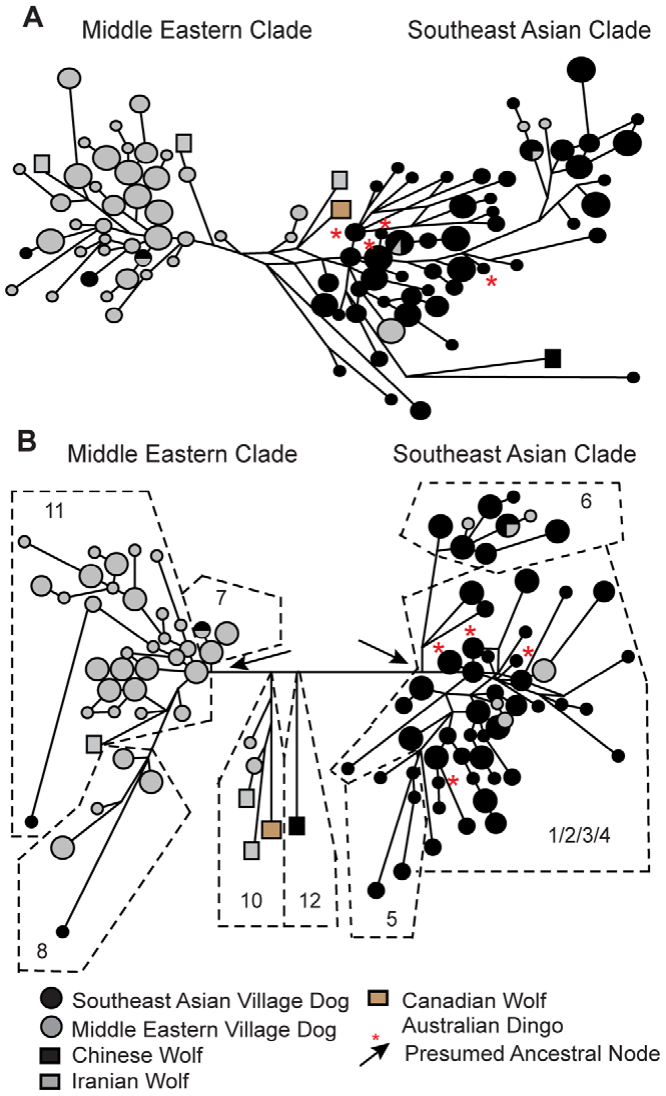
Village dog Y chromosome STR and SNP-STR haplotype networks. Networks of 300 village dog/dingo (circles) and 7 wolf (squares) (a) NRY STR haplotypes and (b) NRY SNP-STR haplotypes, including (a,b) 164 Southeast Asian village dogs/dingoes, 136 Middle Eastern village dogs, 1 Chinese wolf, 3 Iranian wolves, and 3 Canadian wolves. Size of circle is proportional to sample size, except that the largest circle represents 18–50 individuals, and line lengths are proportional to the number of mutational steps. (b) NRY SNP-STR subclades corresponding to numbered SNP haplotypes in Figure 1 are circumscribed by dashed black lines.

The 5 NRY STRs revealed considerably more Y chromosome diversity, including 95 haplotypes in the 300 village dogs and dingoes (and 5 more in the 7 wolves). The diversity of village dog STR haplotypes also was higher than previously found in a much larger sample of breed dogs ([21]; see below). As with the SNP haplotypes, the STR haplotypes formed 2 clades corresponding respectively to Southeast Asia and the Middle East (Figure 2a). Although the positioning of a few of the more distinct STR haplotypes (e.g., >3 mutations from others), including all wolf haplotypes, were discordant with the corresponding SNP haplotypes, this was expected due to homoplasy in the STRs, which limits the phylogentic information contained in more divergent haplotypes. Nevertheless, the similarity in topologies of the STR and SNP networks with respect to the village dogs provided important confirmation that neither topology was overly distorted, respectively, by ascertainment bias associated with the SNP discovery process [39] or homoplasy limiting the extent of divergence revealed by STRs [40].

Combining the two marker types corrected inconsistencies due to ambiguous positioning of distinct STR haplotypes, revealed a greater mutational distance between the Southeast Asian and Middle Eastern clades, equalized the apparent divergence of both dog clades from the basal wolf clade, and revealed that several Middle Eastern village dog haplotypes clustered with the wolf clade, suggestive of recent wolf introgression in Middle Eastern village dogs. The 4 haplotypes exhibited by Australian dingoes clustered more closely with Southeast Asian village dogs, including a basal haplotype shared with some village dogs. The haplotypes of Bali dogs also clustered within the Southeast Asian clade. The haplotypes of Southeast Asian village dogs clustering in the Middle Eastern clade were linked by long branches, indicating ancient derivation. Additionally, the SNP-STR network revealed a diversity of STR haplotypes corresponding to each of the SNP haplotype. Only SNP haplotypes 7 and 11 shared a STR haplotype, indicating that the SNP separating these haplotypes was likely a recent mutation. Thus, the SNP- STR data set provided a powerful basis for assessing ancient vs. recent divergence between village dog populations and for comparing to the haplotypes of breed dogs to infer their proximate origins.

Based on the dog haplotypes of the SNP-STR network (i.e., excluding wolves), each haplotype was rooted to its most proximate common ancestral node (arrows in Figure 2b) and the average number of mutational steps (ρ) to descendant nodes was estimated. These ρ estimates suggested a more recent origin of the Middle Eastern village dog clade (ρ = 3.41 mutations, “95%” CI = 2.0–4.9) than the Southeast Asian Village dog clade (ρ = 6.46 mutations, “95%” CI = 3.8–9.1). The ρ value associated with two clades unique to dogs on Bali was used to calibrate these values. Specifically, ρ = 2.0 (“95%” CI = 0.41–3.59) was estimated for a subclade rooted to haplotype 0d (descendent nodes 0a, 0c, 0e, 0g, 0i, 0k, 1d) and ρ = 1.0 (“95%” CI = 0.12–1.88) for a subclade rooted to haplotype 0f (descendent nodes 0b, 0h, 0j; Tables S8, S9). This produced an average estimated ρ for the population of ρ = 1.5. These values suggest that the Middle Eastern clade was 2–3 times older than the Bali clades and that the Southeast Asian clade was approximately 4 times older than the Bali clades. An isolation time of 3,000 years for the Bali population [18], [27] implies an accumulation of 1 mutation per 2,000 years, yielding an estimated age of the Middle Eastern clade of 6,820 BP (“95%” CI = 3,931–9,709 BP) and an estimated age of the Southeast Asian clade of 12,920 BP (“95%” CI = 7,628–18,212 BP). The corresponding STR haplotype mutation rate, 5.0 * 10^−4^ per year, fell within the expected range for mutation of human Y STRs (e.g., [22]).

### Relationships of modern breeds to village dogs of the Middle East and Southeast Asia

**Figure 3:**
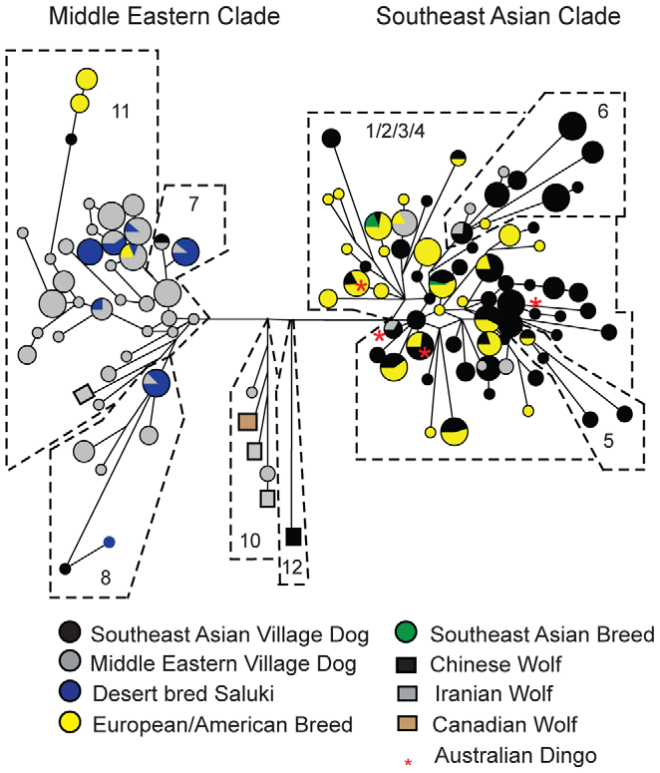
Village and breed dog Y chromosome SNP-STR haplotype networks. Networks of dog (circles) and wolf (squares) NRY SNP-STR haplotypes, including 300 village dogs/dingoes, 124 breed dogs, and 7 wolves. Size of circle is proportional to sample size, except that the larges circle represents 18–50 individuals, and line lengths are proportional to the number of mutational steps. Subclades are numbered corresponding to SNP haplotypes in Figure 1, and are circumscribed by dashed black lines.

We typed 124 male breed dogs at Y chromosome STR and SNP markers, including 62 dogs from putative Western (European, American, Australian) breeds, 3 from 2 East Asian breeds, 31 from a Middle Eastern breed, desert-bred (Bedouin) Salukis from Israel, and 28 of various mixed breeds (Figure 3; Table S10). The Salukis exhibited 11 haplotypes, all of which clustered with Middle Eastern village dogs (Figure S2; Table S10). Six of the 11 haplotypes were shared by Middle Eastern village dogs, possibly reflecting Bedouin reliance on local village dogs for breeding stock. Two beagle haplotypes were in the Middle Eastern clade but clustered with one of the Southeast Asian village dog haplotypes that was 10 mutations from other haplotypes in the Middle Eastern clade, indicating an ancient connection to this clade. Only one haplotype was shared between western breeds (2 boxers and an American pit bull) and Salukis and Middle Eastern village dogs. All other dog haplotypes clustered within the Southeast Asian clade.

**Figure 4:**
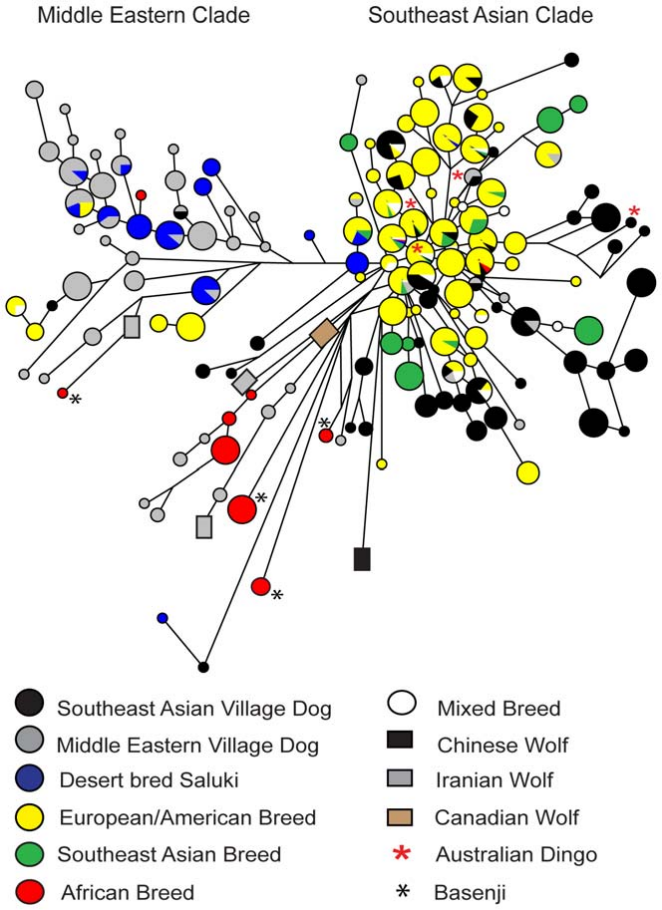
Y chromosome STR haplotype network indicating putative geographic origin of breed dogs. Samples include 428 village dogs, dingoes, and breed dogs genotyped in this study and 818 published breed dog haplotypes [21], color coded according to putative region of breed origin (Table S10). Size of circle is proportional to sample size, except that the largest circle represents 18–50 individuals, and line lengths are proportional to the number of mutational steps.

Lastly, we compared STR haplotypes from a much larger published sample of breed dogs (*n* = 818; [21]) to our village and breed dogs (Figure 4). The placement of the wolf and village dog haplotypes were similar in this network to the previous one based solely on STR types of these dogs (i.e., Figure 2a), suggesting the network accurately depicted close-clustering haplogroups and identified divergent ones, but was unlikely to accurately reflect the deeper phylogeny (e.g., placement of basal nodes of longer branches). As with the 93 European, Southeast Asian, and American breed dogs examined above, the haplotypes (*n* = 60) from these 818 breed dogs clustered primarily in the Southeast Asian village dog clade, including all but 12 European and American breed dogs (Bulldogs, Mastiff, and Jack Russell Terriers) sharing 2 haplotypes (Figure 4; Table S10). Importantly, these 2 haplotypes were highly distinct from others (e.g., similarly to the wolves); therefore, without associated Y-SNP data, the apparent clade-association is inconclusive. Also similarly to our Saluki data, all published desert bred Saluki haplotypes were associated with Middle Eastern clades, as were all 5 Afghan hounds, and 1 Canaan dog, although 2 other Canaan dogs had haplotypes clustering with the Southeast Asian clades (Table S10). Most importantly, the haplotypes of 27 dogs from 3 African breeds were mostly distinct from both Middle Eastern and Southeast Asian clades. The 14 Basenjis were especially distinct based on STR types [21], which is consistent with the haplotype of a previously SNP-typed Basenji (haplotype 9, [25]), which also clustered as distinct from other dogs (Figure 1).

## Discussion

Although molecular genetic approaches can provide powerful tools to study geographic origins of dogs, their value depends on the use of dogs (and/or wolves) that are representative of their indigenous ancestry. Thus, our first question in this study was whether contemporary village dogs in Asia (including parts of Southeast Asia and the Middle East) primarily exhibited indigenous ancestry or, alternatively, whether they reflected admixture among recently created breeds (i.e., mongrels). Second, because breeds are so commonly used to trace ultimate origins (e.g., [12], [14]), we wished to determine geographic origins of these breeds relative to regional indigenous village dog populations. To avoid circularity, we first investigated the roots of village dogs independently of breed dogs. Specifically, we used Y chromosome haplotypes with a sufficiently rapid “half-life” to approximately age clades corresponding to village dog populations in the Middle East and Southeast Asia.

Our findings indicated that Middle Eastern and Southeast Asian village dog populations must have originated either from a common gene pool thousands of years before present or from distinct groups of wolf or wolf-like founders, but are clearly not the product of a post-Victorian expansion of dog breeds. First, the monophyly of Y chromosome clades associated with the two populations, and their long and similar divergence from wolves, suggest that most of the extant Y chromosome diversity evolved after these populations were established. The numbers of mutations separating haplotypes and their ancestral nodes in each of these clades provided a measure of time to most recent common ancestor, which also reflected the minimum time since separation. The time estimates, while necessarily imprecise, were qualitatively robust because they were based on calibration to known-age indigenous, insular dog populations of Bali and Australia. These Island populations were known to be founded on the order of 3,000–5,000 years BP, based on archaeological evidence [17], [18], [27] and confirmed with mtDNA both for Bali dogs (this study) and dingoes [28]. Thus, extant Middle Eastern and Southeast Asian village dog patrilines clearly reflect a deep divergence reaching at least as far back as 10,000–16,000 years. Moreover, comparison to the previously published Y chromosome STR [21] and SNP [25] haplotypes of African breeds indicate these reflect at least one more divergent paternal lineage of dogs not present in Asia. This finding emphasizes the need for expanding Y chromosome analysis to African village dogs, such as those previously investigated at mtDNA and nuclear markers [20] to explore the age and origins of these dogs as well. Even with respect to Asia, uncertainty in the SNP haplotype mutation rates along with unknown ascertainment biases prevent putting an upper limit on the divergence time estimate pending additional Y chromosome sequencing. However, mtDNA evidence suggests that these populations are probably not much more than 16,000 years divergent [9], [14].

Having established that village dogs used in this study reflect indigenous populations, it was possible to compare ancestry of breeds with respect to these indigenous populations. Although we could not trace modern breeds to a precise location of origin because we did not sample village dogs from a large intervening portion of Asia, the considerable genetic distance between these village dog populations, even if ends of a continuum, enabled inference about the relative regional affinities. As expected, Middle Eastern breeds clustered with Middle Eastern village dogs and East Asian breeds clustered with Southeast Asian village dogs. However, putative European and American breeds also clustered in the Southeast Asian clade, which ran counter to expectations. It would not have been surprising to find some Eastern influence in Western breeds, as many breeds developed during the Victorian dog-fancy era either were initiated from East Asian stock [41]–[43], or were admixed with it at some stage of breed formation [44]. However, the near complete lack of Middle Eastern haplotypes in Western breeds was unexpected given the relative proximity of Europe to the Middle East relative to East Asia. The most parsimonious interpretation of these findings would seem to be that modern European breed dogs are overwhelmingly derived from recently imported exotic stock and not reflective of ancient indigenous ancestry. This interpretation is also supported by findings in ancient DNA studies in Europe and the America's, which have uniformly found discontinuities between ancient and modern dogs, indicating relatively recent replacements of historical dog populations with post-Victorian breed dogs [4], [15], [16].

If Western breeds do not derive from their putative regions of origin, this warrants reconsideration of some previous conclusions about dog origins. In particular, Savolainen et al. [6] and Pang et al. [14] interpreted lower observed mtDNA diversity and greater proportional composition of UTs in Europe than the Middle East and, in turn, the Middle East than Southeast Asia, to support the hypothesis that dogs initially must have spread from east to west across southern Eurasia and that dogs were least likely to have originated in Europe [6], [14]. Clearly, if the dogs used in those studies to represent Europe derive from exotic sources, as our findings and others suggest, it would seem premature to exclude Europe as a viable candidate for the site (or one of the sites) of dog origins, especially in light of other evidence in its favor (e.g., [7], [9], [45]).

On the other hand, our findings with both mtDNA and Y chromosome analyses provided strong confirmation of higher diversity previously observed in Southeast Asia than the Middle East [6], [14]. This conclusion has been one of two principal pieces of evidence supporting the Southeast Asian-origins hypothesis (the other being morphological similarities with Chinese wolves [13]). However, the initial samples showing higher mtDNA diversity in Southeast Asia than the Middle East were skewed, including a relatively small number (n = 37) of village dogs from the Middle East, potentially biased by differing compositions of breed and village dogs [20]. Nonetheless, the present study added hundreds more village dogs to both regions and provided near identical estimates both of mtDNA diversity in general and in terms of the proportional composition of UTs, and the pattern held both for localized sampling sites and the entire regions. This confirmation is important for a second reason as well. Because our Southeast Asian sample was drawn from further south in continental Asia and near-Island Southeast Asia, whereas the previous one was from a smaller region of South China [14], our analysis effectively expanded the size of the Southeast Asian region over which dog evolution studies are likely to be fruitful. Because of the possibility of bias due to sampling a larger region in the Southeast Asia (which, in our case, also was a structured population) than the Middle East [e.g., 20], we also looked within localized sampling sites and, again, found the highest genetic diversity in Southeast Asian dogs. The exception was from dogs on Bali, the southernmost Island sampled, and known to have been long-isolated from mainland Southeast Asia. Numbers of accumulated mutations between ancestral and descendent nodes (i.e., ρ estimates) also were consistent with an older Southeast Asian than Middle Eastern Y chromosome clade, possibly a reflection of effective population size more so than population age, but, nevertheless, of higher diversity in the Southeast. Thus, it seems well-supported based on both matrilineal and patrilineal markers that extant dogs of Southeast Asia, over an even larger region than that identified by Pang et al. [14] and including near Island Southeast Asia, harbor more genetic diversity than the Middle East. Although these findings do not constitute proof that dogs originated in Southeast Asia [12], [20], [45], they clearly indicate continuity with a very ancient dog population in that region and, therefore, that it likely played an important role in the evolution of modern dogs.

### Implications for future research

Up to now, use of ancient DNA approaches to the study of dog origins have been limited to mtDNA, which has been most useful in showing that modern dogs do not necessarily reflect the ancient ones inhabiting those same regions [4], [15],[16], but see [9]. However, the low resolution of mtDNA examined in those studies, combined with the apparently rapid expansion of early dogs, has prevented strong inferences about dog origins. In contrast, the distinct geographically associated Y-chromosome village dog haplogroups observed in this study provide a potentially strong basis for reconstructing geographic origins of progressively more ancient samples. The Middle Eastern and Southeast Asian village dog Y chromosome haplotypes can be further augmented through expanded sampling in Africa, Central and northern Eurasia, and the Americas, which will provide a powerful frame of reference against which ancient samples can be compared to reconstruct dog migrations through time and, hence, to better illuminate their origins, whether ultimately multiple or singular. Although nuclear DNA (i.e., including Y chromosome) is more difficult than mtDNA to study in ancient samples, it is technically feasible, has been done previously in other species [46], and doing so would seem to hold considerable promise to answer previously unanswerable questions about dog origins.

## Supporting Information

Figure S1 **Sampling locations throughout the Middle East and Southeast Asia.** Yangtze River is indicated by blue line. Israeli desert-bred Saluki (1; yellow circle, *n* = 45), Iranian village dogs (grey circles: 2 = Kazerun, *n* = 22; 3 = Shiraz, *n* = 180; 4 = Kerman, *n* = 31), Southeast Asian village dogs (black circles; 5 = Thailand, *n* = 57; 6 = Taiwan, *n* = 40; 7 = Philippines, *n* = 26; 8 = Brunei, *n* = 27; 9 = Bali, *n* = 97; 10 = Australian Dingo, *n* = 15).
(TIF)

Figure S2 **Y chromosome SNP-STR haplotype network depicting haplotype names.** Network of dog (circles) and wolf (squares) NRY SNP-STR haplotypes, including 300 village dogs, 124 breed dogs, and 7 wolves. Haplotype names are beside their respective haplotype. Size of circle is proportional to sample size, except that the larges circle represents 18–50 individuals. NRY SNP-STR subclades corresponding to numbered SNP haplotypes in Figure 1 (in main text) are circumscribed by dashed black lines.
(TIF)

Table S1 **List of Sequenom PCR primers developed for SNP loci, which correspond to Natanaelsson et al. 2006^a^.**
(DOC)

Table S2 **Sequenom SNP extension primer sequences and expected extension products, developed for SNP loci, which correspond to Natanaelsson et al. 2006^a^.**
(DOC)

Table S3 **Frequency of 402 bp mtDNA haplotypes of village dogs sampled in 7 populations.** Haplotypes are named as the lowest-numbered previously named synonymous 582 bp haplotype Savolainen et al. 2002^a^ and Pang et al. 2009^b^ or are novel in this study (haplotype names beginning with “V”); see Table S4 for additional information on novel haplotypes.
(DOCX)

Table S4 **Seventeen novel mtDNA haplotypes, location of sample, and number of substitutions differing from the nearest published haplotype.** Sequences were deposited in Genbank (Accession Nos. HQ287728–HQ287744, respectively, in order presented below).
(DOC)

Table S5 **Number of individuals (** ***n*** **), number (No.) of mtDNA haplotypes, and rarified haplotype richness (corrected for differing sample sizes to** ***n*** **= 10) for 402 bp mtDNA D-loop haplotypes of village dog sampling locations in the Middle East and Southeast Asia.**
(DOC)

Table S6 **Y chromosome SNP haplotypes as resolved from 11 “Ydog” loci (Natanaelsson et al. 2005^a^).** Haplotypes 1–9 have been described previously in terms of these and additional loci and haplotypes 10–12, named in this study, were represented previously as unsampled, unnamed nodes.
(DOCX)

Table S7 **Imputed Y-SNP haplotypes corresponding to the 23 (of 120 total) Y-STR haplotypes for which 1 or more of the 11 SNPs failed (indicated by “-”) or for which no SNP genotyping was attempted.** All but one incomplete SNP haplotype corresponded to the same failed locus and the same haplotype ambiguity (7 or 11), suggesting a mutation in the priming region associated with this clade. The SNP haplotypes are indicated in Table S6.
(DOCX)

Table S8 **Allelic composition of NRY STR haplotypes and corresponding SNP haplotypes.** An asterix identifies a SNP haplotype with one or more positions imputed (see Table S7). Previously published haplotypes are named the same as by Bannasch et al. 2005^a^.
(DOCX)

Table S9 **Frequency of NRY STR and SNP haplotypes observed in Middle Eastern and Southeast Asian village dog populations, Australian Dingoes, breed dogs (including published ones^a^), and gray wolves.** An asterix identifies a SNP haplotype with one or more positions imputed (see Table S7).
(DOCX)

Table S10 **Breed, number (No.) of individuals, putative region of breed origin^a^, breed class, and haplotype name of STR haplotypes included in NRY STR and SNP-STR analyses.** Parentheses indicate individuals and haplotypes found in breed dogs that were genotyped in the present study and used in SNP-STR analysis; otherwise entries refer to published data^b^.
(DOCX)
