# Supplementary figures and images for: Phylogenetic Distinctiveness of Middle Eastern and Southeast Asian Village Dog Y Chromosomes Illuminates Dog Origins
Source: PLoS One. 2011 Dec 14;6(12):e28496. doi: 10.1371/journal.pone.0028496 (PMC3237445; doi:10.1371/journal.pone.0028496)

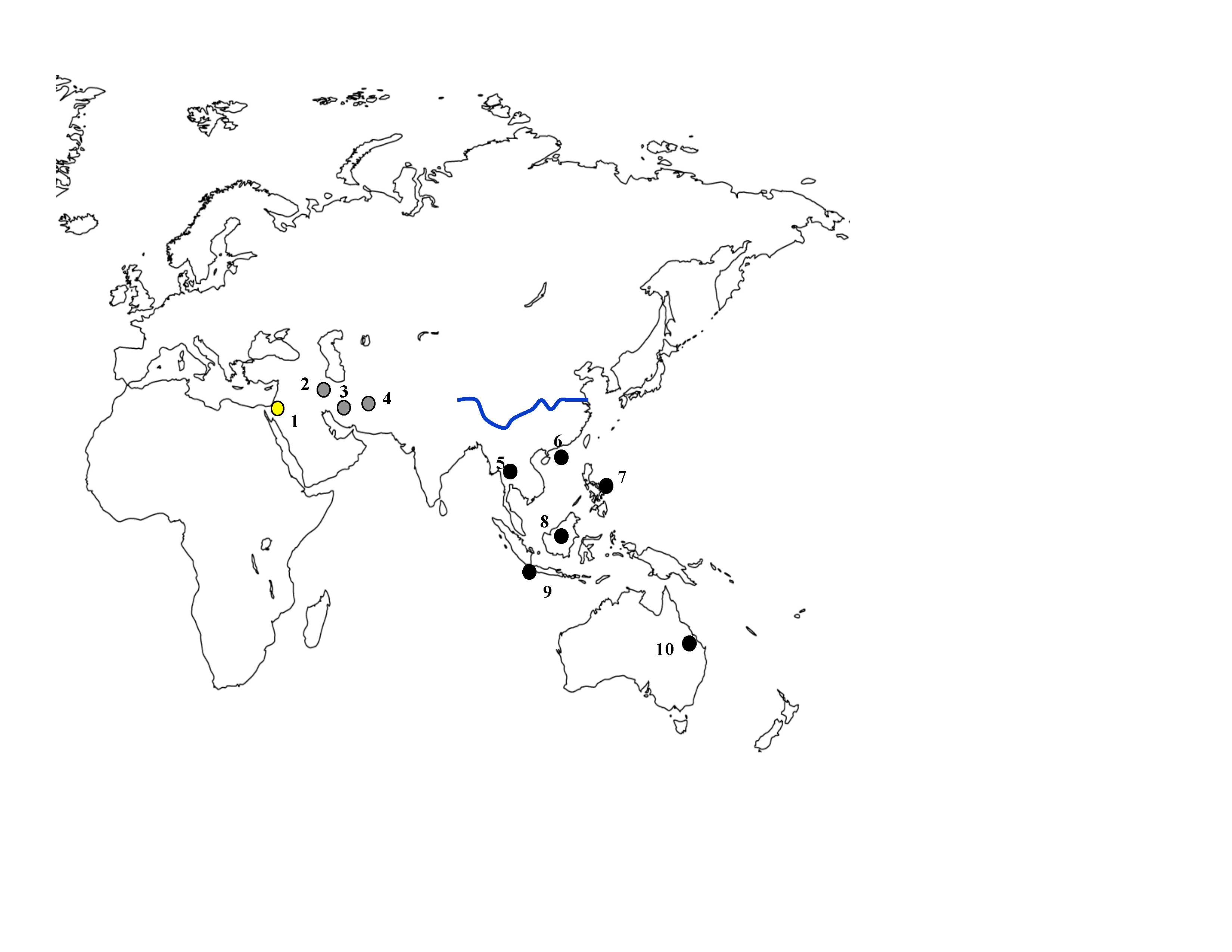

Supplement: Figure S1 — Sampling locations throughout the Middle East and Southeast Asia. Yangtze River is indicated by blue line. Israeli desert-bred Saluki (1; yellow circle, n = 45), Iranian village dogs (grey circles: 2 = Kazerun, n = 22; 3 = Shiraz, n = 180; 4 = Kerman, n = 31), Southeast Asian village dogs (black circles; 5 = Thailand, n = 57; 6 = Taiwan, n = 40; 7 = Philippines, n = 26; 8 = Brunei, n = 27; 9 = Bali, n = 97; 10 = Australian Dingo, n = 15). (TIF) [file pone.0028496.s001.tif]

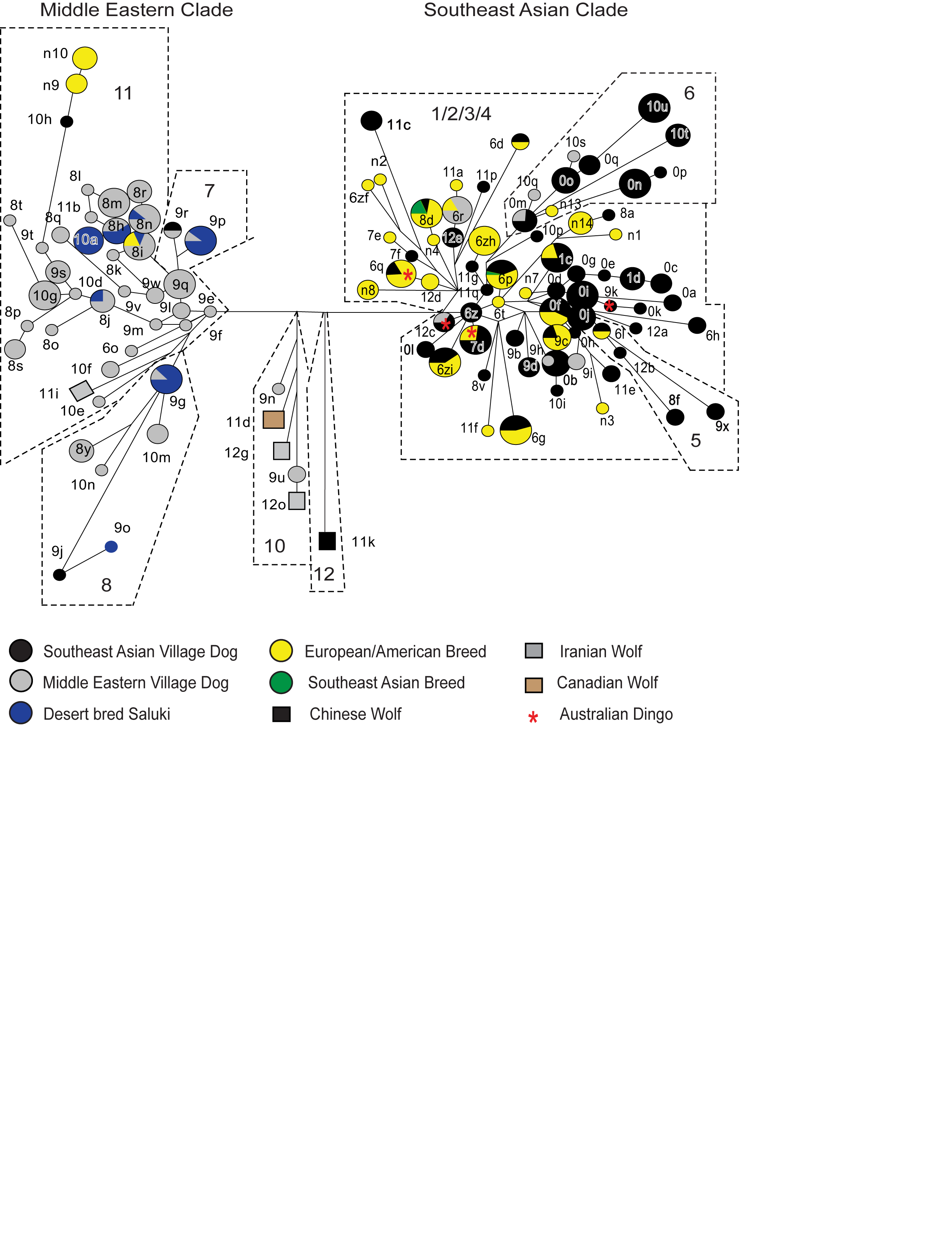

Supplement: Figure S2 — Y chromosome SNP-STR haplotype network depicting haplotype names. Network of dog (circles) and wolf (squares) NRY SNP-STR haplotypes, including 300 village dogs, 124 breed dogs, and 7 wolves. Haplotype names are beside their respective haplotype. Size of circle is proportional to sample size, except that the larges circle represents 18–50 individuals. NRY SNP-STR subclades corresponding to numbered SNP haplotypes in Figure 1 (in main text) are circumscribed by dashed black lines. (TIF) [file pone.0028496.s002.tif]
