# Supplementary material for: Phylogenetic Distinctiveness of Middle Eastern and Southeast Asian Village Dog Y Chromosomes Illuminates Dog Origins
Source: PLoS One. 2011 Dec 14;6(12):e28496. doi: 10.1371/journal.pone.0028496 (PMC3237445; doi:10.1371/journal.pone.0028496)
Supplement: Table S1 — List of Sequenom PCR primers developed for SNP loci, which correspond to Natanaelsson et al. 2006a. (DOC) [file pone.0028496.s003.doc]

Table S1. List of Sequenom PCR primers developed for SNP loci, which correspond to Natanaelsson et al. 2006^a^.

| SNP_ID | PCR primer^b^ | PCR primer |
| --- | --- | --- |
| Ydog_20 | acgttggatggtaggacaaactacagagaa | acgttggatgctccttttacacattttctc |
| Ydog_21 | acgttggatgattagttcttggggcacctg | acgttggatgtagcactctgagatcaggac |
| Ydog_28_1_1 | acgttggatgggtaacttactgtgagaaag | acgttggatgcaccaaaactctaaatcagc |
| Ydog_28_1_2 | acgttggatgcaccaaaactctaaatcagc | acgttggatgggtaacttactgtgagaaag |
| Ydog_28_2 | acgttggatgcactttacacattgttagaag | acgttggatggtgatcaacttgaatctatcc |
| Ydog_29_part2 | acgttggatgatttgaacactaatatgttg | acgttggatggttcaaaagtacttaacagac |
| Ydog_30 | acgttggatgtttctatttatgtttgttgc | acgttggatgagccgaaagcggcactaaac |
| Ydog_B_part2 | acgttggatgggagtgtggtaggcttatag | acgttggatgtgcagcccacagtttttagc |
| Ydog_G1_part1 | acgttggatggtagtccttaattatctggg | acgttggatgggaatccctttgcaaaggac |
| Ydog_G1_part2 | acgttggatgggacaaaaccggatttccac | acgttggatgtatatcttctttggtactg |
| Ydog_N | acgttggatgcaaacctttaatcatctggg | acgttggatggaaaccaaatcaaaccaaac |

^a^Natanaelsson C, Oskarsson MCR, Angleby H, Lundeberg J, Kirkness E, et al. (2006)

Dog Y chromosomal DNA sequence: identification, sequencing and SNP discovery. BMC

Genet 7: 45.

^b^All primers have generic 10mer tag “ACGTTGGATG” added to their sequence.
