# Supplementary material for: Phylogenetic Distinctiveness of Middle Eastern and Southeast Asian Village Dog Y Chromosomes Illuminates Dog Origins
Source: PLoS One. 2011 Dec 14;6(12):e28496. doi: 10.1371/journal.pone.0028496 (PMC3237445; doi:10.1371/journal.pone.0028496)
Supplement: Table S2 — Sequenom SNP extension primer sequences and expected extension products, developed for SNP loci, which correspond to Natanaelsson et al. 2006a. (DOC) [file pone.0028496.s004.doc]

Table S2. Sequenom SNP extension primer sequences and expected extension products, developed for SNP loci, which correspond to Natanaelsson et al. 2006^a^.

| SNP_ID | Extension Primer Direction | Extension Primer Sequence | Extension Product 1 Call | Extension Product 1 Sequence | Extension Product 2 Call | Extension Product 2 Sequence |
| --- | --- | --- | --- | --- | --- | --- |
| Ydog_20 | F | ttacacattttctcttccatt | A | ttacacattttctcttccatta | G | ttacacattttctcttccattg |
| Ydog_21 | R | accaagagtcagatacttaa | G | accaagagtcagatacttaac | A | accaagagtcagatacttaat |
| Ydog_28_1_1 | F | gcatcttttaaaagctcgt | A | gcatcttttaaaagctcgta | G | gcatcttttaaaagctcgtg |
| Ydog_28_1_2 | R | actgtgagaaagaataaagtattta | C | actgtgagaaagaataaagtatttag | A | actgtgagaaagaataaagtatttat |
| Ydog_28_2 | R | caaaaaaggcaagggtc | G | caaaaaaggcaagggtcc | A | caaaaaaggcaagggtct |
| Ydog_29_part2 | R | aaactcaaattaggaatatctttt | T | aaactcaaattaggaatatctttta | A | aaactcaaattaggaatatcttttt |
| Ydog_30 | F | aaccgctgagccacc | C | aaccgctgagccaccc | T | aaccgctgagccacct |
| Ydog_B_part2 | F | gtttttagcttctccaaaatga | C | gtttttagcttctccaaaatgac | T | gtttttagcttctccaaaatgat |
| Ydog_G1_part1 | F | ttggtctctcagctact | C | ttggtctctcagctactc | T | ttggtctctcagctactt |
| Ydog_G1_part2 | F | tttggtactgtatattatctctc | C | tttggtactgtatattatctctcc | A | tttggtactgtatattatctctca |
| Ydog_N | R | ccaaatcaaaccaaacataacc | G | ccaaatcaaaccaaacataaccc | C | ccaaatcaaaccaaacataaccg |

^a^Natanaelsson C, Oskarsson MCR, Angleby H, Lundeberg J, Kirkness E, et al. (2006)

Dog Y chromosomal DNA sequence: identification, sequencing and SNP discovery. BMC

Genet 7: 45.
