# Supplementary material for: Phylogenetic Distinctiveness of Middle Eastern and Southeast Asian Village Dog Y Chromosomes Illuminates Dog Origins
Source: PLoS One. 2011 Dec 14;6(12):e28496. doi: 10.1371/journal.pone.0028496 (PMC3237445; doi:10.1371/journal.pone.0028496)
Supplement: Table S3 — Frequency of 402 bp mtDNA haplotypes of village dogs sampled in 7 populations. Haplotypes are named as the lowest-numbered previously named synonymous 582 bp haplotype Savolainen et al. 2002a and Pang et al. 2009b or are novel in this study (haplotype names beginning with “V”); see Table S4 for additional information on novel haplotypes. (DOCX) [file pone.0028496.s005.docx]

Table S3. Frequency of 402 bp mtDNA haplotypes of village dogs sampled in 7 populations. Haplotypes are named as the lowest-numbered previously named synonymous 582 bp haplotype Savolainen et al. 2002^a^, and Pang et al. 2009^b^ or are novel in this study (haplotype names beginning with “V”); see Table S4 for additional information on novel haplotypes.

| Haplotype | Taiwan | Thailand | Bali | Philippines | Dingo | Brunei | Iran |
| --- | --- | --- | --- | --- | --- | --- | --- |
| A1 | -- | 2 | -- | -- | -- | -- | -- |
| A11 | 5 | 13 | -- | -- | -- | 2 | 43 |
| A116 | -- | -- | 11 | -- | -- | -- | -- |
| A120 | -- | 1 | -- | -- | -- | -- | -- |
| A135 | -- | -- | -- | -- | -- | -- | 2 |
| A14 | -- | -- | -- | -- | -- | -- | 2 |
| A145 | -- | -- | 1 | -- | -- | -- | -- |
| A152 | -- | -- | 2 | -- | -- | -- | 2 |
| A16 | 8 | 5 | 2 | 1 | -- | 2 | 11 |
| A18 | 8 | 4 | 4 | 2 | -- | 1 | 16 |
| A19 | 5 | -- | -- | 4 | -- | -- | 44 |
| A22 | -- | -- | -- | -- | -- | -- | 3 |
| A26 | 1 | 1 | -- | -- | -- | -- | 4 |
| A27 | 1 | 1 | -- | 4 | -- | -- | 5 |
| A28 | -- | -- | -- | -- | -- | -- | 2 |
| A3 | -- | 3 | 1 | -- | -- | 1 | 3 |
| A30 | -- | -- | -- | -- | -- | -- | 2 |
| A44 | -- | 2 | 1 | -- | -- | -- | -- |
| A49 | -- | -- | 5 | -- | -- | -- | 1 |
| A75 | -- | -- | 51 | -- | -- | -- | -- |
| B1 | 5 | 8 | 2 | 3 | -- | -- | 27 |
| B11 | -- | -- | -- | -- | -- | -- | 1 |
| B12 | -- | -- | -- | -- | -- | -- | 1 |
| B23 | 2 | -- | 1 | -- | -- | -- | -- |
| B6 | 2 | 2 | -- | 1 | -- | -- | 11 |
| C1 | 1 | -- | 3 | 5 | -- | 2 | 7 |
| C3 | -- | 3 | -- | -- | -- | -- | 5 |
| C5 | 1 | -- | -- | -- | -- | -- | -- |
| D6 | -- | -- | -- | -- | -- | -- | 1 |
| DIN15 | -- | -- | -- | -- | 1 | -- | -- |
| DIN20 | -- | -- | -- | -- | 8 | -- | -- |
| DIN3 | -- | -- | -- | -- | 1 | -- | -- |
| E1 | -- | -- | 3 | -- | -- | -- | -- |
| V6 | -- | -- | 2 | -- | -- | -- | -- |
| V7 | -- | -- | 2 | -- | -- | -- | -- |
| V9 | -- | -- | 1 | -- | -- | -- | -- |
|  |  |  |  |  |  |  |  |
| Haplotype | Taiwan | Thailand | Bali | Philippines | Dingo | Brunei | Iran |
| V10 | -- | -- | 1 | -- | -- | -- | -- |
| V11 | -- | -- | 1 | -- | -- | -- | -- |
| V129 | **--** | **1** | **--** | **--** | **--** | **--** | **--** |
| V14 | -- | 3 | -- | -- | -- | -- | -- |
| V157 | 1 | -- | -- | -- | -- | -- | -- |
| V225 | -- | -- | -- | -- | -- | -- | 1 |
| V234 | -- | -- | -- | -- | -- | -- | 1 |
| V302 | -- | -- | -- | -- | -- | -- | 1 |
| V33 | -- | -- | -- | -- | -- | -- | 1 |
| V369 | -- | -- | -- | -- | -- | -- | 1 |
| V372 | -- | -- | -- | -- | -- | -- | 1 |
| V403 | -- | -- | -- | -- | -- | -- | 1 |
| V491 | -- | -- | -- | -- | -- | 4 | -- |
| V506 | -- | -- | -- | -- | -- | 1 | -- |
| V514 | -- | -- | -- | 1 | -- | -- | -- |
| V516 | -- | -- | -- | 1 | -- | -- | -- |
| V525 | -- | -- | -- | 2 | -- | -- | -- |
| VT9 | -- | 1 | -- | -- | -- | -- | -- |
| Total | 40 | 50 | 94 | 24 | 10 | 13 | 200 |

^a^Savolainen P, Zhang Y, Luo J, Lundeberg J, Leitner T (2002) Genetic evidence for an East

Asian origin of domestic dogs. Science 298: 1610-1613.

^b^Pang J-F, Kluetsch C, Zou X-J, Zhang A-B, Luo L-Y, et al. (2009) mtDNA data indicate a

single origin for dogs south of Yangtze River, less than 16,300 years ago, from numerous wolves. Mol Biol Evol 26: 2849-2864.
