# Supplementary material for: Phylogenetic Distinctiveness of Middle Eastern and Southeast Asian Village Dog Y Chromosomes Illuminates Dog Origins
Source: PLoS One. 2011 Dec 14;6(12):e28496. doi: 10.1371/journal.pone.0028496 (PMC3237445; doi:10.1371/journal.pone.0028496)
Supplement: Table S4 — Seventeen novel mtDNA haplotypes, location of sample, and number of substitutions differing from the nearest published haplotype. Sequences were deposited in Genbank (Accession Nos. HQ287728–HQ287744, respectively, in order presented below). (DOC) [file pone.0028496.s006.doc]

Table S4. Seventeen novel mtDNA haplotypes, location of sample, and number of substitutions differing from the nearest published haplotype. Sequences were deposited in Genbank (Accession Nos. HQ287728-- HQ287744, respectively, in order presented below).

| Novel Haplotype | Location | No. of substitutions | Nearest Haplotype^a,b^ |
| --- | --- | --- | --- |
| Southeast Asia |  |  |  |
| V10 | Bali | 1 | A13, A123, A89, (A11), A142 |
| V11 | Bali | 1 | A141, A116 |
| V6 | Bali | 1 | A141, A116 |
| V7 | Bali | 1 | A141, A116 |
| V9 | Bali | 2 | A13, A123, A89, (A11), A142 |
| V506 | Brunei | 1 | V491 |
| V514 | Philippines | 1 | A13, A123, A89, (A11), A142 |
| V516 | Philippines | 1 | B8, B7, B33, (B6), B10 |
| V525 | Philippines | 1 | (A18), A94, A20 |
| V129 | Taiwan | 1 | A114, A44 |
| V14 | Thailand | 1 | B13, B3, B21, (B1), B15, B16, B5, B22, B5 |
| VT9 | Thailand | 1 | A140, A138, A5, (A3), A9, A8, A7, A130 |
| Middle East |  |  |  |
| V225 | Iran | 1 | A62, A158, A6 |
| V302 | Iran | 1 | (C1), C15, C2 |
| V369 | Iran | 1 | A13, A123, A89, (A11), A142 |
| V33 | Iran | 1 | B13, B3, B21, (B1), B15, B16, B5, B22, B5 |
| SA403 | Saluki | 1 | V157, C7 |

^a^Our village dog haplotypes were based on 402 bp instead of 582 bp as per Savolainen et al. 2002^c^, and Pang et al. 2009^d^, therefore multiple 582 bp haplotypes sometimes correspond to a single 402 bp haplotype.

^b^Haplotypes in parentheses represent UTs (Universal Types) as per Pang et al. 2009.

^c^Savolainen P, Zhang Y, Luo J, Lundeberg J, Leitner T (2002) Genetic evidence for an East

Asian origin of domestic dogs. Science 298: 1610-1613.

^d^Pang J-F, Kluetsch C, Zou X-J, Zhang A-B, Luo L-Y, et al. (2009) mtDNA data indicate a

single origin for dogs south of Yangtze River, less than 16,300 years ago, from numerous wolves. Mol Biol Evol 26: 2849-2864.
