# Supplementary material for: Phylogenetic Distinctiveness of Middle Eastern and Southeast Asian Village Dog Y Chromosomes Illuminates Dog Origins
Source: PLoS One. 2011 Dec 14;6(12):e28496. doi: 10.1371/journal.pone.0028496 (PMC3237445; doi:10.1371/journal.pone.0028496)
Supplement: Table S5 — Number of individuals ( n ), number (No.) of mtDNA haplotypes, and rarified haplotype richness (corrected for differing sample sizes to n = 10) for 402 bp mtDNA D-loop haplotypes of village dog sampling locations in the Middle East and Southeast Asia. (DOC) [file pone.0028496.s007.doc]

Table S5. Number of individuals (*n*), number (No.) of mtDNA haplotypes, and rarified haplotype richness (corrected for differing sample sizes to *n* = 10) for 402 bp mtDNA D-loop haplotypes of village dog sampling locations in the Middle East and Southeast Asia.

| Population | *n* | No. Haplotypes | Haplotype Richness |
| --- | --- | --- | --- |
| Middle East |  |  |  |
| Kazerun, Iran | 21 | 8 | 5.02 |
| Shiraz, Iran | 149 | 23 | 5.47 |
| Kerman, Iran | 30 | 11 | 5.13 |
| Southeast Asia |  |  |  |
| Phuket, Thailand | 50 | 15 | 6.23 |
| Chiayi City, Taiwan | 40 | 12 | 5.70 |
| Quezon City, Philipines | 24 | 9 | 5.75 |
| Bandar Seri Begawan,  Brunei Darussalam | 13 | 7 | 5.50 |
| Bali, Indonesia | 94 | 18 | 4.34 |
| Fraser Island (Dingo),  Australia | 10 | 3 | 2.00 |
