# Supplementary material for: Phylogenetic Distinctiveness of Middle Eastern and Southeast Asian Village Dog Y Chromosomes Illuminates Dog Origins
Source: PLoS One. 2011 Dec 14;6(12):e28496. doi: 10.1371/journal.pone.0028496 (PMC3237445; doi:10.1371/journal.pone.0028496)
Supplement: Table S6 — Y chromosome SNP haplotypes as resolved from 11 “Ydog” loci (Natanaelsson et al. 2005a). Haplotypes 1–9 have been described previously in terms of these and additional loci and haplotypes 10–12, named in this study, were represented previously as unsampled, unnamed nodes. (DOCX) [file pone.0028496.s008.docx]

Table S6. Y chromosome SNP haplotypes as resolved from 11 “Ydog” loci (Natanaelsson et al. 2005^a^). Haplotypes 1—9 have been described previously in terms of these and additional loci and haplotypes 10-12, named in this study, were represented previously as unsampled, unnamed nodes.

|  | Ydog locus | | | | | | | | | | |
| --- | --- | --- | --- | --- | --- | --- | --- | --- | --- | --- | --- |
| SNP Clade | 20 | 21 | 28a | 28b | 28c | 29 part2 | 30 | B  part2 | G part1a | G part2 | N |
| reference | A | G | A | A | G | A | C | C | T | C | C |
| 1/2/3/4 | . | . | . | . | . | . | . | . | . | . | . |
| 5 | . | . | . | . | . | T | . | . | . | . | . |
| 6 | G | . | . | . | . | . | . | . | . | . | G |
| 7 | . | . | G | C | A | . | . | . | C | A | . |
| 8 | . | . | G | C | A | . | T | . | . | A | . |
| 9 | . | A | G | C | . | . | . | T | . | A | . |
| 10 | . | A | G | C | . | . | . | . | . | A | . |
| 11 | . | . | G | C | A | . | . | . | . | A | . |
| 12 | . | . | G | C | . | . | . | . | . | A | . |

^a^ Natanaelsson C, Oskarsson MCR, Angleby H, Lundeberg J, Kirkness E, et al. (2006)

Dog Y chromosomal DNA sequence: identification, sequencing and SNP discovery. BMC

Genet 7: 45.
