# Supplementary material for: Phylogenetic Distinctiveness of Middle Eastern and Southeast Asian Village Dog Y Chromosomes Illuminates Dog Origins
Source: PLoS One. 2011 Dec 14;6(12):e28496. doi: 10.1371/journal.pone.0028496 (PMC3237445; doi:10.1371/journal.pone.0028496)
Supplement: Table S7 — Imputed Y-SNP haplotypes corresponding to the 23 (of 120 total) Y-STR haplotypes for which 1 or more of the 11 SNPs failed (indicated by “-”) or for which no SNP genotyping was attempted. All but one incomplete SNP haplotype corresponded to the same failed locus and the same haplotype ambiguity (7 or 11), suggesting a mutation in the priming region associated with this clade. The SNP haplotypes are indicated in Table S6. (DOCX) [file pone.0028496.s009.docx]

Table S7. Imputed Y-SNP haplotypes corresponding to the 23 (of 120 total) Y-STR haplotypes for which 1 or more of the 11 SNPs failed (indicated by “-”) or for which no SNP genotyping was attempted. All but one incomplete SNP haplotype corresponded to the same failed locus and the same haplotype ambiguity (7 or 11), suggesting a mutation in the priming region associated with this clade. The SNP haplotypes are indicated in Table S6.

| STR haplotype name | No. dogs | Incomplete SNP haplotype | Possible corresponding SNP haplotypes | STR haplotypes differing by 1^a^ stepwise mutation | Corresp. SNP haplotype name ^b^ |
| --- | --- | --- | --- | --- | --- |
| 10d | 1 | AGGCAACC-AC | 7, 11 | 10c,10g,8j,9 | 11 |
| 10e | 1 | AGGCAACC-AC | 7, 11 | 10a,10f | 11 |
| 10f | 1 | AGGCAACC-AC | 7, 11 | 10e | 11 |
| 4c | 1 | AGGCAACC-AC | 7, 11 | 4d,8h,8l | 11 |
| 8o | 1 | AGGCA-CC-AC | 7, 11 | 8n,8p | 11 |
| 9f | 1 | -GGCAACC-AC | 7, 11 | 4k,9e,9m | 11 |
| 9k | 1 | -GAAG-CCTCC | 1/2/3/4, 5, 6 | 0i,0k, | 1/2/3/4 |
| 9l | 2 | AGGCAACC-AC | 7, 11 | 10b,9e,9m | 11 |
| 9m | 1 | AGGCAACC-AC | 7, 11 | 9f,9l | 11 |
| 9p | 1 | AGGCAACC-AC | 7, 11 | 10a,10b,4j,9v | 11 |
| 9s | 4 | AGGCAACC-AC | 7, 11 | 10d,9t | 11 |
| 9t | 1 | AGGCAACC-AC | 7, 11 | 9s | 11 |
| 9v | 1 | AGGCAACC-AC | 7, 11 | 4h,9w | 11 |
| n10 | 4 | AGGCAACC-AC | 7, 11 | n9 | 11 |
| n9 | 3 | AGGCAACC-AC | 7, 11 | 10h,n10 | 11 |
| 0i | 9 | not attempted | -- | 0d,9k | 1/2/3/4 |
| 0d | 2 | not attempted | -- | 0e,0i,6v,n7 | 1/2/3/4 |
| 0c | 2 | not attempted | -- | 11e,1d,9c,n7 | 1/2/3/4 |
| 9h | 1 | not attempted | -- | 6zb,9i | 1/2/3/4 |
| 9o* | 1 | not attempted | -- | 9j **^a^** | 8 |
| 9w | 2 | not attempted | -- | 10b,9v | 11 |
| 9z* | 1 | not attempted | -- | 10a,10b,10c,10f,8i **^a^** | 11 |
| 10a | 6 | not attempted | -- | 10e,3g,8h,9p | 7, 11 |

**^a^** Haplotypes 9o and 9z differered by 2 stepwise mutations from nearest STR haplotypes

**^b^** These are the SNP haplotypes as imputed for purposes of network construction based on clustering with STR haplotypes
