# Supplementary material for: Phylogenetic Distinctiveness of Middle Eastern and Southeast Asian Village Dog Y Chromosomes Illuminates Dog Origins
Source: PLoS One. 2011 Dec 14;6(12):e28496. doi: 10.1371/journal.pone.0028496 (PMC3237445; doi:10.1371/journal.pone.0028496)
Supplement: Table S8 — Allelic composition of NRY STR haplotypes and corresponding SNP haplotypes. An asterix identifies a SNP haplotype with one or more positions imputed (see Table S7). Previously published haplotypes are named the same as by Bannasch et al. 2005a. (DOCX) [file pone.0028496.s010.docx]

Table S8. Allelic composition of NRY STR haplotypes and corresponding SNP haplotypes. An asterix identifies a SNP haplotype with one or more positions imputed (see Table S7). Previously published haplotypes are named the same as by Bannasch et al. 2005^a^.

| Haplotype | 79.2 | 79.3 | 990.35.4 | ms34CA | ms41b | SNP Haplotype |
| --- | --- | --- | --- | --- | --- | --- |
| 0a | 128 | 128 | 126 | 172 | 222 | 1/2/3/4 |
| 0b | 134 | 122 | 128 | 172 | 220 | 1/2/3/4 |
| 0c | 134 | 124 | 128 | 172 | 222 | 1/2/3/4* |
| 0d | 134 | 126 | 126 | 172 | 222 | 1/2/3/4* |
| 0e | 134 | 126 | 126 | 172 | 224 | 1/2/3/4 |
| 0f | 134 | 126 | 128 | 172 | 220 | 1/2/3/4 |
| 0g | 134 | 126 | 128 | 172 | 224 | 1/2/3/4 |
| 0h | 134 | 126 | 130 | 172 | 220 | 1/2/3/4 |
| 0i | 134 | 128 | 126 | 172 | 222 | 1/2/3/4* |
| 0j | 134 | 128 | 128 | 172 | 220 | 1/2/3/4 |
| 0k | 134 | 132 | 126 | 172 | 222 | 1/2/3/4 |
| 0l | 136 | 128 | 126 | 176 | 216 | 1/2/3/4 |
| 0m | 138 | 128 | 126 | 172 | 214 | 6 |
| 0n | 138 | 132 | 126 | 172 | 210 | 6 |
| 0o | 138 | 132 | 126 | 172 | 214 | 6 |
| 0p | 138 | 134 | 126 | 172 | 210 | 6 |
| 0q | 138 | 134 | 126 | 172 | 214 | 6 |
| 10a | 134 | 134 | 126 | 176 | 214 | 11* |
| 10d | 134 | 134 | 126 | 176 | 220 | 11 |
| 10e | 134 | 134 | 126 | 178 | 214 | 11 |
| 10f | 134 | 134 | 126 | 178 | 216 | 11 |
| 10g | 134 | 134 | 126 | 178 | 220 | 11 |
| 10h | 134 | 134 | 126 | 178 | 222 | 7 |
| 10i | 136 | 122 | 128 | 172 | 220 | 1/2/3/4 |
| 10m | 136 | 130 | 126 | 180 | 216 | 8 |
| 10n | 136 | 130 | 126 | 180 | 220 | 8 |
| 10p | 138 | 124 | 126 | 172 | 214 | 1/2/3/4 |
| 10q | 138 | 126 | 126 | 172 | 214 | 6 |
| 10s | 138 | 132 | 128 | 172 | 214 | 6 |
| 10t | 138 | 134 | 126 | 172 | 216 | 6 |
| 10u | 140 | 136 | 126 | 170 | 214 | 6 |
| 11a | 134 | 122 | 128 | 172 | 214 | 1/2/3/4 |
| 11b | 134 | 136 | 126 | 176 | 212 | 11 |
| 11c | 134 | 124 | 128 | 168 | 210 | 1/2/3/4 |
| 11d | 134 | 124 | 128 | 174 | 218 | 10 |
| Haplotype | 79.2 | 79.3 | 990.35.4 | ms34CA | ms41b | SNP Haplotype |
| 11e | 134 | 124 | 130 | 172 | 222 | 1/2/3/4 |
| 11f | 136 | 124 | 128 | 172 | 216 | 1/2/3/4 |
| 11g | 136 | 126 | 126 | 172 | 214 | 1/2/3/4 |
| 11i | 134 | 130 | 126 | 182 | 214 | 11 |
| 11k | 134 | 132 | 134 | 170 | 212 | 12 |
| 11p | 136 | 124 | 128 | 172 | 214 | 1/2/3/4 |
| 11q | 136 | 128 | 126 | 172 | 214 | 1/2/3/4 |
| 12a | 132 | 124 | 126 | 172 | 220 | 1/2/3/4 |
| 12b | 132 | 128 | 126 | 174 | 220 | 5 |
| 12c | 132 | 126 | 126 | 172 | 216 | 1/2/3/4 |
| 12d | 134 | 126 | 126 | 172 | 214 | 1/2/3/4 |
| 12e | 134 | 126 | 128 | 172 | 214 | 1/2/3/4 |
| 12g | 132 | 126 | 128 | 174 | 220 | 10 |
| 12o | 134 | 132 | 128 | 174 | 224 | 10 |
| 1c | 134 | 122 | 126 | 172 | 220 | 1/2/3/4 |
| 1d | 134 | 124 | 128 | 172 | 224 | 1/2/3/4 |
| 3a | 132 | 132 | 126 | 172 | 218 | -- |
| 3b | 132 | 132 | 126 | 172 | 220 | -- |
| 3c | 134 | 122 | 128 | 172 | 212 | -- |
| 3d | 134 | 122 | 128 | 172 | 214 | -- |
| 3e | 134 | 126 | 128 | 172 | 210 | -- |
| 3g | 134 | 134 | 126 | 174 | 214 | -- |
| 3h | 138 | 130 | 126 | 172 | 210 | -- |
| 3i | 134 | 126 | 126 | 176 | 214 | -- |
| 4a | 134 | 126 | 134 | 174 | 228 | -- |
| 4b | 134 | 130 | 126 | 176 | 210 | -- |
| 4c | 134 | 136 | 126 | 176 | 212 | -- |
| 4d | 134 | 136 | 128 | 176 | 212 | -- |
| 4e | 136 | 130 | 134 | 174 | 216 | -- |
| 4f | 136 | 130 | 134 | 184 | 216 | -- |
| 4g | 138 | 132 | 126 | 182 | 216 | -- |
| 4h | 134 | 132 | 128 | 176 | 212 | -- |
| 4i | 134 | 134 | 128 | 176 | 212 | -- |
| 4j | 134 | 132 | 126 | 176 | 212 | -- |
| 4k | 134 | 126 | 126 | 176 | 218 | -- |
| 6a | 134 | 132 | 128 | 172 | 218 | -- |
| 6b | 128 | 126 | 126 | 174 | 212 | -- |
| 6c | 128 | 126 | 128 | 172 | 212 | -- |
| 6d | 128 | 126 | 128 | 172 | 214 | 1/2/3/4* |
| Haplotype | 79.2 | 79.3 | 990.35.4 | ms34CA | ms41b | SNP Haplotype |
| 6e | 128 | 126 | 126 | 172 | 216 | -- |
| 6f | 128 | 126 | 126 | 174 | 216 | -- |
| 6g | 128 | 126 | 128 | 172 | 216 | 1/2/3/4 |
| 6h | 128 | 126 | 126 | 172 | 220 | 1/2/3/4 |
| 6i | 128 | 128 | 126 | 172 | 218 | -- |
| 6j | 132 | 126 | 126 | 172 | 216 | -- |
| 6k | 132 | 128 | 126 | 172 | 212 | -- |
| 6l | 132 | 130 | 126 | 172 | 220 | 5 |
| 6m | 134 | 122 | 126 | 172 | 214 | -- |
| 6o | 134 | 124 | 126 | 176 | 216 | 11 |
| 6p | 134 | 124 | 126 | 172 | 218 | 1/2/3/4 |
| 6q | 134 | 126 | 126 | 172 | 214 | 1/2/3/4 |
| 6r | 134 | 126 | 128 | 172 | 214 | 1/2/3/4 |
| 6s | 134 | 126 | 128 | 172 | 216 | -- |
| 6t | 134 | 126 | 126 | 172 | 218 | 1/2/3/4 |
| 6u | 134 | 126 | 128 | 172 | 218 | 1/2/3/4 |
| 6v | 134 | 126 | 126 | 172 | 220 | -- |
| 6w | 134 | 126 | 126 | 174 | 212 | -- |
| 6x | 134 | 126 | 128 | 172 | 212 | -- |
| 6y | 134 | 128 | 126 | 172 | 210 | -- |
| 6z | 134 | 128 | 126 | 172 | 216 | -- |
| 6za | 134 | 128 | 126 | 172 | 218 | -- |
| 6zb | 134 | 128 | 128 | 172 | 218 | -- |
| 6zc | 134 | 128 | 126 | 178 | 222 | -- |
| 6zd | 134 | 128 | 126 | 178 | 224 | -- |
| 6ze | 136 | 124 | 128 | 172 | 216 | -- |
| 6zf | 136 | 126 | 126 | 172 | 210 | 1/2/3/4 |
| 6zg | 136 | 126 | 126 | 172 | 212 | -- |
| 6zh | 136 | 126 | 126 | 172 | 214 | 1/2/3/4 |
| 6zi | 136 | 126 | 126 | 172 | 216 | 1/2/3/4 |
| 6zj | 138 | 124 | 126 | 172 | 210 | -- |
| 7a | 132 | 126 | 126 | 172 | 212 | -- |
| 7b | 134 | 122 | 126 | 172 | 212 | -- |
| 7c | 134 | 124 | 126 | 176 | 214 | -- |
| 7d | 134 | 126 | 126 | 172 | 216 | 1/2/3/4 |
| 7e | 134 | 126 | 126 | 172 | 212 | 1/2/3/4 |
| 7f | 134 | 128 | 126 | 172 | 212 | -- |
| 8a | 128 | 122 | 126 | 172 | 218 | 1/2/3/4 |
| 8d | 132 | 124 | 126 | 172 | 214 | 1/2/3/4 |
| Haplotype | 79.2 | 79.3 | 990.35.4 | ms34CA | ms41b | SNP Haplotype |
| 8f | 132 | 126 | 126 | 174 | 224 | 5 |
| 8h | 134 | 136 | 126 | 176 | 214 | 11 |
| 8i | 134 | 136 | 126 | 176 | 216 | 11 |
| 8j | 134 | 134 | 126 | 176 | 218 | 11 |
| 8k | 134 | 136 | 128 | 176 | 216 | 11 |
| 8l | 134 | 138 | 126 | 176 | 212 | 11 |
| 8m | 134 | 138 | 126 | 176 | 214 | 11 |
| 8n | 134 | 138 | 126 | 176 | 216 | 11 |
| 8o | 134 | 138 | 126 | 176 | 218 | 11 |
| 8p | 134 | 138 | 126 | 176 | 220 | 11 |
| 8q | 134 | 138 | 128 | 176 | 214 | 11 |
| 8r | 134 | 140 | 126 | 176 | 216 | 11 |
| 8s | 134 | 140 | 126 | 176 | 220 | 11 |
| 8t | 134 | 140 | 126 | 178 | 220 | 11 |
| 8v | 134 | 122 | 128 | 172 | 216 | 1/2/3/4 |
| 8y | 134 | 124 | 126 | 180 | 220 | 8 |
| 9c | 134 | 124 | 128 | 172 | 220 | 1/2/3/4 |
| 9d | 134 | 124 | 130 | 172 | 218 | 1/2/3/4 |
| 9e | 134 | 128 | 126 | 176 | 216 | 11 |
| 9f | 134 | 128 | 126 | 176 | 218 | 11 |
| 9g | 134 | 128 | 126 | 180 | 216 | 8 |
| 9h | 134 | 128 | 128 | 174 | 218 | 1/2/3/4* |
| 9i | 134 | 128 | 128 | 174 | 220 | 1/2/3/4 |
| 9j | 134 | 128 | 142 | 180 | 216 | 8 |
| 9k | 134 | 130 | 126 | 172 | 222 | 1/2/3/4 |
| 9l | 134 | 130 | 126 | 176 | 216 | 11 |
| 9m | 134 | 130 | 126 | 176 | 218 | 11 |
| 9n | 134 | 130 | 130 | 174 | 216 | 10 |
| 9o | 134 | 130 | 142 | 180 | 218 | 8* |
| 9p | 134 | 132 | 126 | 176 | 214 | 7 |
| 9q | 134 | 132 | 126 | 176 | 216 | 7 and 11 |
| 9r | 134 | 132 | 126 | 176 | 218 | 7 |
| 9s | 134 | 132 | 126 | 176 | 220 | 11 |
| 9t | 134 | 132 | 126 | 176 | 222 | 11 |
| 9u | 134 | 132 | 128 | 174 | 222 | 10 |
| 9v | 134 | 132 | 128 | 176 | 214 | 11 |
| 9w | 134 | 132 | 128 | 176 | 216 | 11* |
| 9x | 134 | 134 | 126 | 172 | 218 | 5 |
| 9z | 134 | 134 | 126 | 174 | 216 | -- |
| Haplotype | 79.2 | 79.3 | 990.35.4 | ms34CA | ms41b | SNP Haplotype |
| n1 | 128 | 124 | 126 | 174 | 218 | 1/2/3/4 |
| n10 | 134 | 136 | 126 | 178 | 224 | 11 |
| n13 | 138 | 128 | 126 | 172 | 212 | 6 |
| n14 | 128 | 124 | 126 | 172 | 218 | 1/2/3/4 |
| n2 | 132 | 124 | 126 | 172 | 210 | 1/2/3/4 |
| n3 | 132 | 124 | 130 | 172 | 222 | 1/2/3/4 |
| n4 | 132 | 126 | 126 | 172 | 214 | 1/2/3/4 |
| n7 | 134 | 124 | 126 | 172 | 222 | 1/2/3/4 |
| n8 | 134 | 124 | 126 | 174 | 214 | 1/2/3/4 |
| n9 | 134 | 134 | 126 | 178 | 224 | 11 |

^a^Bannasch D, Bannasch M, Ryun J, Famula T, Pedersen N (2005) Y chromosome haplotype analysis in purebred dogs. Mamm Genome 16: 273-280.
