# Supplementary material for: Phylogenetic Distinctiveness of Middle Eastern and Southeast Asian Village Dog Y Chromosomes Illuminates Dog Origins
Source: PLoS One. 2011 Dec 14;6(12):e28496. doi: 10.1371/journal.pone.0028496 (PMC3237445; doi:10.1371/journal.pone.0028496)
Supplement: Table S9 — Frequency of NRY STR and SNP haplotypes observed in Middle Eastern and Southeast Asian village dog populations, Australian Dingoes, breed dogs (including published onesa), and gray wolves. An asterix identifies a SNP haplotype with one or more positions imputed (see Table S7). (DOCX) [file pone.0028496.s011.docx]

Table S9. Frequency of NRY STR and SNP haplotypes observed in Middle Eastern and Southeast Asian village dog populations, Australian Dingoes, breed dogs (including published ones^a^), and gray wolves. An asterix identifies a SNP haplotype with one or more positions imputed (see Table S7).

| STR haplotype | SNP haplotype | Bali | Brunei | Iran | Thailand | Taiwan | Philippines | Israeli Saluki | Dingo | Breed | Published Breed^a^ | Wolf |
| --- | --- | --- | --- | --- | --- | --- | --- | --- | --- | --- | --- | --- |
| 0a | 1/2/3/4 | 2 | -- | -- | -- | -- | -- | -- | -- | -- | -- | -- |
| 0b | 1/2/3/4 | 5 | -- | -- | -- | -- | -- | -- | -- | -- | -- | -- |
| 0c | 1/2/3/4* | 3 | -- | -- | -- | -- | -- | -- | -- | -- | -- | -- |
| 0d | 1/2/3/4* | 2 | -- | -- | -- | -- | -- | -- | -- | -- | -- | -- |
| 0e | 1/2/3/4 | 2 | -- | -- | -- | -- | -- | -- | -- | -- | -- | -- |
| 0f | 1/2/3/4 | 4 | -- | -- | -- | -- | -- | -- | -- | 3 | -- | -- |
| 0g | 1/2/3/4 | 1 | -- | -- | -- | -- | -- | -- | -- | -- | -- | -- |
| 0h | 1/2/3/4 | 3 | -- | -- | -- | -- | -- | -- | -- | -- | -- | -- |
| 0i | 1/2/3/4* | 9 | -- | -- | -- | -- | -- | -- | -- | -- | -- | -- |
| 0j | 1/2/3/4 | 5 | -- | -- | -- | -- | -- | -- | -- | -- | -- | -- |
| 0k | 1/2/3/4 | 1 | -- | -- | -- | -- | -- | -- | -- | -- | -- | -- |
| 0l | 1/2/3/4 | 2 | -- | -- | -- | -- | -- | -- | -- | -- | -- | -- |
| 0m | 6 | 4 | -- | 1 | -- | -- | -- | -- | -- | -- | -- | -- |
| 0n | 6 | 18 | -- | -- | 1 | -- | -- | -- | -- | -- | -- | -- |
| 0o | 6 | 5 | -- | -- | -- | -- | -- | -- | -- | -- | -- | -- |
| 0p | 6 | 1 | -- | -- | -- | -- | -- | -- | -- | -- | -- | -- |
| 0q | 6 | 1 | 2 | -- | -- | -- | -- | -- | -- | -- | -- | -- |
| 10a | 11* | -- | -- | -- | -- | -- | -- | 6 | -- | -- | -- | -- |
| 10d | 11 | -- | -- | 1 | -- | -- | -- | -- | -- | -- | -- | -- |
| 10e | 11 | -- | -- | 1 | -- | -- | -- | -- | -- | -- | -- | -- |
| 10f | 11 | -- | -- | 2 | -- | -- | -- | -- | -- | -- | -- | -- |
| 10g | 11 | -- | -- | 8 | -- | -- | -- | -- | -- | -- | -- | -- |
| STR haplotype | SNP haplotype | Bali | Brunei | Iran | Thailand | Taiwan | Philippines | Israeli Saluki | Dingo | Breed | Published Breed^a^ | Wolf |
| 10h | 7 | -- | -- | -- | -- | 1 | -- | -- | -- | -- | -- | -- |
| 10i | 1/2/3/4 | -- | -- | -- | -- | 1 | -- | -- | -- | -- | -- | -- |
| 10m | 8 | -- | -- | 3 | -- | -- | -- | -- | -- | -- | -- | -- |
| 10n | 8 | -- | -- | 1 | -- | -- | -- | -- | -- | -- | -- | -- |
| 10p | 1/2/3/4 | -- | 1 | -- | -- | -- | -- | -- | -- | -- | -- | -- |
| 10q | 6 | -- | -- | 1 | -- | -- | -- | -- | -- | -- | -- | -- |
| 10s | 6 | -- | -- | 1 | -- | -- | -- | -- | -- | -- | -- | -- |
| 10t | 6 | -- | 5 | -- | -- | -- | -- | -- | -- | -- | -- | -- |
| 10u | 6 | -- | -- | -- | 9 | -- | -- | -- | -- | -- | -- | -- |
| 11a | 1/2/3/4 | -- | -- | -- | -- | -- | -- | -- | -- | 1 | -- | -- |
| 11b | 11 | -- | -- | 1 | -- | -- | -- | -- | -- | -- | -- | -- |
| 11c | 1/2/3/4 | -- | -- | -- | -- | -- | 3 | -- | -- | -- | -- | -- |
| 11d | 10 | -- | -- | -- | -- | -- | -- | -- | -- | -- | -- | 3 |
| 11e | 1/2/3/4 | -- | -- | -- | -- | -- | 2 | -- | -- | -- | -- | -- |
| 11f | 1/2/3/4 | -- | -- | -- | -- | -- | -- | -- | -- | 1 | -- | -- |
| 11g | 1/2/3/4 | -- | -- | 1 | 1 | -- | -- | -- | -- | -- | -- | -- |
| 11i | 11 | -- | -- | -- | -- | -- | -- | -- | -- | -- | -- | 1 |
| 11k | 12 | -- | -- | -- | -- | -- | -- | -- | -- | -- | -- | 1 |
| 11p | 1/2/3/4 | -- | -- | -- | -- | -- | 1 | -- | -- | -- | -- | -- |
| 11q | 1/2/3/4 | -- | -- | -- | -- | -- | 1 | -- | -- | -- | -- | -- |
| 12a | 1/2/3/4 | -- | -- | -- | -- | -- | 1 | -- | -- | -- | -- | -- |
| 12b | 5 | -- | -- | -- | -- | -- | 1 | -- | -- | -- | -- | -- |
| 12c | 1/2/3/4 | -- | -- | 1 | -- | -- | -- | -- | 2 | -- | -- | -- |
| 12d | 1/2/3/4 | -- | -- | -- | -- | -- | -- | -- | -- | 2 | -- | -- |
| 12e | 1/2/3/4 | -- | -- | -- | 1 | -- | 2 | -- | -- | -- | -- | -- |
| 12g | 10 | -- | -- | -- | -- | -- | -- | -- | -- | -- | -- | 1 |
| STR haplotype | SNP haplotype | Bali | Brunei | Iran | Thailand | Taiwan | Philippines | Israeli Saluki | Dingo | Breed | Published Breed^a^ | Wolf |
| 12o | 10 | -- | -- | -- | -- | -- | -- | -- | -- | -- | -- | 1 |
| 1c | 1/2/3/4 | -- | -- | -- | 5 | 2 | 1 | -- | -- | 2 | -- | -- |
| 1d | 1/2/3/4 | 2 | -- | -- | -- | -- | -- | -- | -- | -- | -- | -- |
| 3a | -- | -- | -- | -- | -- | -- | -- | -- | -- | -- | 7 | -- |
| 3b | -- | -- | -- | -- | -- | -- | -- | -- | -- | -- | 3 | -- |
| 3c | -- | -- | -- | -- | -- | -- | -- | -- | -- | -- | 11 | -- |
| 3d | -- | -- | -- | -- | -- | -- | -- | -- | -- | -- | 2 | -- |
| 3e | -- | -- | -- | -- | -- | -- | -- | -- | -- | -- | 5 | -- |
| 3g | -- | -- | -- | -- | -- | -- | -- | -- | -- | -- | 3 | -- |
| 3h | -- | -- | -- | -- | -- | -- | -- | -- | -- | -- | 16 | -- |
| 3i | -- | -- | -- | -- | -- | -- | -- | -- | -- | -- | 5 | -- |
| 4a | -- | -- | -- | -- | -- | -- | -- | -- | -- | -- | 8 | -- |
| 4b | -- | -- | -- | -- | -- | -- | -- | -- | -- | -- | 3 | -- |
| 4c | -- | -- | -- | -- | -- | -- | -- | -- | -- | -- | 1 | -- |
| 4d | -- | -- | -- | -- | -- | -- | -- | -- | -- | -- | 11 | -- |
| 4e | -- | -- | -- | -- | -- | -- | -- | -- | -- | -- | 2 | -- |
| 4f | -- | -- | -- | -- | -- | -- | -- | -- | -- | -- | 3 | -- |
| 4g | -- | -- | -- | -- | -- | -- | -- | -- | -- | -- | 1 | -- |
| 4h | -- | -- | -- | -- | -- | -- | -- | -- | -- | -- | 1 | -- |
| 4i | -- | -- | -- | -- | -- | -- | -- | -- | -- | -- | 2 | -- |
| 4j | -- | -- | -- | -- | -- | -- | -- | -- | -- | -- | 3 | -- |
| 4k | -- | -- | -- | -- | -- | -- | -- | -- | -- | -- | 1 | -- |
| 6a | -- | -- | -- | -- | -- | -- | -- | -- | -- | -- | 5 | -- |
| 6b | -- | -- | -- | -- | -- | -- | -- | -- | -- | -- | 3 | -- |
| 6c | -- | -- | -- | -- | -- | -- | -- | -- | -- | -- | 1 | -- |
| 6d | 1/2/3/4* | -- | 1 | -- | -- | -- | -- | -- | -- | 1 | 24 | -- |
| STR haplotype | SNP haplotype | Bali | Brunei | Iran | Thailand | Taiwan | Philippines | Israeli Saluki | Dingo | Breed | Published Breed^a^ | Wolf |
| 6e | -- | -- | -- | -- | -- | -- | -- | -- | -- | -- | 25 | -- |
| 6f | -- | -- | -- | -- | -- | -- | -- | -- | -- | -- | 23 | -- |
| 6g | 1/2/3/4 | -- | 3 | -- | 2 | -- | -- | -- | -- | 6 | 4 | -- |
| 6h | 1/2/3/4 | 1 | -- | -- | -- | 1 | -- | -- | -- | -- | 15 | -- |
| 6i | -- | -- | -- | -- | -- | -- | -- | -- | -- | -- | 1 | -- |
| 6j | -- | -- | -- | -- | -- | -- | -- | -- | -- | -- | 1 | -- |
| 6k | -- | -- | -- | -- | -- | -- | -- | -- | -- | -- | 18 | -- |
| 6l | 5 | -- | -- | 1 | -- | -- | -- | -- | -- | 1 | 5 | -- |
| 6m | -- | -- | -- | -- | -- | -- | -- | -- | -- | -- | 1 | -- |
| 6o | 11 | -- | -- | 1 | -- | -- | -- | -- | -- | -- | 1 | -- |
| 6p | 1/2/3/4 | -- | 2 | 1 | 4 | 5 | 1 | -- | -- | -- | 36 | -- |
| 6q | 1/2/3/4 | -- | -- | -- | -- | -- | -- | -- | 1 | 5 | 36 | -- |
| 6r | 1/2/3/4 | -- | -- | 5 | -- | -- | -- | -- | -- | 1 | 28 | -- |
| 6s | -- | -- | -- | -- | -- | -- | -- | -- | -- | -- | 1 | -- |
| 6t | 1/2/3/4 | -- | -- | -- | -- | -- | -- | -- | -- | 16 | 6 | -- |
| 6u | 1/2/3/4 | -- | -- | -- | 2 | -- | -- | -- | -- | -- | 11 | -- |
| 6v | -- | -- | -- | -- | -- | -- | -- | -- | -- | -- | 2 | -- |
| 6w | -- | -- | -- | -- | -- | -- | -- | -- | -- | -- | 1 | -- |
| 6x | -- | -- | -- | -- | -- | -- | -- | -- | -- | -- | 11 | -- |
| 6y | -- | -- | -- | -- | -- | -- | -- | -- | -- | -- | 2 | -- |
| 6z | -- | -- | -- | -- | 1 | -- | -- | -- | -- | -- | 14 | -- |
| 6za | -- | -- | -- | -- | -- | -- | -- | -- | -- | 1 | 4 | -- |
| 6zb | -- | -- | -- | -- | -- | -- | -- | -- | -- | -- | 2 | -- |
| 6zc | -- | -- | -- | -- | -- | -- | -- | -- | -- | -- | 9 | -- |
| 6zd | -- | -- | -- | -- | -- | -- | -- | -- | -- | -- | 3 | -- |
| 6ze | -- | -- | -- | -- | -- | -- | -- | -- | -- | -- | 5 | -- |
| STR haplotype | SNP haplotype | Bali | Brunei | Iran | Thailand | Taiwan | Philippines | Israeli Saluki | Dingo | Breed | Published Breed^a^ | Wolf |
| 6zf | 1/2/3/4 | -- | -- | -- | -- | -- | -- | -- | -- | 1 | 1 | -- |
| 6zg | -- | -- | -- | -- | -- | -- | -- | -- | -- | -- | 27 | -- |
| 6zh | 1/2/3/4 | -- | -- | -- | -- | -- | -- | -- | -- | 7 | 87 | -- |
| 6zi | 1/2/3/4 | -- | 1 | -- | -- | 1 | 2 | -- | -- | 6 | 2 | -- |
| 6zj | -- | -- | -- | -- | -- | -- | -- | -- | -- | -- | 1 | -- |
| 7a | -- | -- | -- | -- | -- | -- | -- | -- | -- | -- | 71 | -- |
| 7b | -- | -- | -- | -- | -- | -- | -- | -- | -- | -- | 12 | -- |
| 7c | -- | -- | -- | -- | -- | -- | -- | -- | -- | -- | 38 | -- |
| 7d | 1/2/3/4 | -- | 3 | -- | 1 | 3 | -- | -- | 1 | 3 | 119 | -- |
| 7e | 1/2/3/4 | -- | -- | -- | -- | -- | -- | -- | -- | 1 | 62 | -- |
| 7f | -- | -- | -- | -- | 1 | -- | -- | -- | -- | -- | 12 | -- |
| 8a | 1/2/3/4 | -- | -- | -- | -- | 1 | -- | -- | -- | -- | -- | -- |
| 8d | 1/2/3/4 | -- | 1 | -- | -- | -- | -- | -- | -- | 10 | -- | -- |
| 8f | 5 | -- | -- | -- | -- | 2 | -- | -- | -- | -- | -- | -- |
| 8h | 11 | -- | -- | 2 | -- | -- | -- | 3 | -- | -- | -- | -- |
| 8i | 11 | -- | -- | 10 | -- | -- | -- | 3 | -- | 3 | -- | -- |
| 8j | 11 | -- | -- | 3 | -- | -- | -- | 1 | -- | -- | -- | -- |
| 8k | 11 | -- | -- | 1 | -- | -- | -- | -- | -- | -- | -- | -- |
| 8l | 11 | -- | -- | 1 | -- | -- | -- | -- | -- | -- | -- | -- |
| 8m | 11 | -- | -- | 7 | -- | -- | -- | -- | -- | -- | -- | -- |
| 8n | 11 | -- | -- | 15 | -- | -- | -- | 2 | -- | -- | -- | -- |
| 8o | 11 | -- | -- | 1 | -- | -- | -- | -- | -- | -- | -- | -- |
| 8p | 11 | -- | -- | 1 | -- | -- | -- | -- | -- | -- | -- | -- |
| 8q | 11 | -- | -- | 2 | -- | -- | -- | -- | -- | -- | -- | -- |
| 8r | 11 | -- | -- | 4 | -- | -- | -- | -- | -- | -- | -- | -- |
| 8s | 11 | -- | -- | 3 | -- | -- | -- | -- | -- | -- | -- | -- |
| STR haplotype | SNP haplotype | Bali | Brunei | Iran | Thailand | Taiwan | Philippines | Israeli Saluki | Dingo | Breed | Published Breed^a^ | Wolf |
| 8t | 11 | -- | -- | 1 | -- | -- | -- | -- | -- | -- | -- | -- |
| 8v | 1/2/3/4 | -- | -- | -- | -- | 1 | -- | -- | -- | -- | -- | -- |
| 8y | 8 | -- | -- | 4 | -- | -- | -- | -- | -- | -- | -- | -- |
| 9c | 1/2/3/4 | -- | -- | -- | 1 | -- | -- | -- | -- | 4 | -- | -- |
| 9d | 1/2/3/4 | -- | 2 | -- | -- | -- | 1 | -- | -- | -- | -- | -- |
| 9e | 11 | -- | -- | 1 | -- | -- | -- | -- | -- | -- | -- | -- |
| 9f | 11 | -- | -- | 1 | -- | -- | -- | -- | -- | -- | -- | -- |
| 9g | 8 | -- | -- | 1 | -- | -- | -- | 7 | -- | -- | -- | -- |
| 9h | 1/2/3/4* | -- | -- | 1 | -- | -- | -- | -- | -- | -- | -- | -- |
| 9i | 1/2/3/4 | -- | -- | 2 | -- | -- | -- | -- | -- | -- | -- | -- |
| 9j | 8 | -- | -- | -- | 1 | -- | -- | -- | -- | -- | -- | -- |
| 9k | 1/2/3/4 | -- | -- | -- | -- | -- | -- | -- | 1 | -- | -- | -- |
| 9l | 11 | -- | -- | 2 | -- | -- | -- | -- | -- | -- | -- | -- |
| 9m | 11 | -- | -- | 1 | -- | -- | -- | -- | -- | -- | -- | -- |
| 9n | 10 | -- | -- | 1 | -- | -- | -- | -- | -- | -- | -- | -- |
| 9o | 8* | -- | -- | -- | -- | -- | -- | 1 | -- | -- | -- | -- |
| 9p | 7 | -- | -- | 1 | -- | -- | -- | 8 | -- | -- | -- | -- |
| 9q | 7 and 11 | -- | -- | 28 | -- | -- | -- | -- | -- | -- | -- | -- |
| 9r | 7 | -- | -- | 1 | 1 | -- | -- | -- | -- | -- | -- | -- |
| 9s | 11 | -- | -- | 4 | -- | -- | -- | -- | -- | -- | -- | -- |
| 9t | 11 | -- | -- | 1 | -- | -- | -- | -- | -- | -- | -- | -- |
| 9u | 10 | -- | -- | 2 | -- | -- | -- | -- | -- | -- | -- | -- |
| 9v | 11 | -- | -- | 1 | -- | -- | -- | -- | -- | -- | -- | -- |
| 9w | 11* | -- | -- | 2 | -- | -- | -- | -- | -- | -- | -- | -- |
| 9x | 5 | -- | -- | -- | -- | 2 | -- | -- | -- | -- | -- | -- |
| 9z | -- | -- | -- | 1 | -- | -- | -- | -- | -- | -- | -- | -- |
| STR haplotype | SNP haplotype | Bali | Brunei | Iran | Thailand | Taiwan | Philippines | Israeli Saluki | Dingo | Breed | Published Breed^a^ | Wolf |
| n1 | 1/2/3/4 | -- | -- | -- | -- | -- | -- | -- | -- | 1 | -- | -- |
| n10 | 11 | -- | -- | -- | -- | -- | -- | -- | -- | 4 | -- | -- |
| n13 | 6 | -- | -- | -- | -- | -- | -- | -- | -- | 1 | -- | -- |
| n14 | 1/2/3/4 | -- | -- | -- | -- | -- | -- | -- | -- | 4 | -- | -- |
| n2 | 1/2/3/4 | -- | -- | -- | -- | -- | -- | -- | -- | 1 | -- | -- |
| n3 | 1/2/3/4 | -- | -- | -- | -- | -- | -- | -- | -- | 1 | -- | -- |
| n4 | 1/2/3/4 | -- | -- | -- | -- | -- | -- | -- | -- | 1 | -- | -- |
| n7 | 1/2/3/4 | -- | -- | -- | -- | -- | -- | -- | -- | 1 | -- | -- |
| n8 | 1/2/3/4 | -- | -- | -- | -- | -- | -- | -- | -- | 3 | -- | -- |
| n9 | 11 | -- | -- | -- | -- | -- | -- | -- | -- | 3 | -- | -- |
| Total | n/a | 71 | 21 | 136 | 31 | 20 | 16 | 31 | 5 | 95 | 818 | 7 |

^a^Bannasch D, Bannasch M, Ryun J, Famula T, Pedersen N (2005). Y chromosome haplotype analysis in purebred dogs. Mamm Genome 16: 273-280. Note: SNP haplotypes were not determined for these published breed samples and when recorded on the same row indicate the SNP haplotype corresponding to a different sample with the same STR haplotype.
