# Supplementary material for: Phylogenetic Distinctiveness of Middle Eastern and Southeast Asian Village Dog Y Chromosomes Illuminates Dog Origins
Source: PLoS One. 2011 Dec 14;6(12):e28496. doi: 10.1371/journal.pone.0028496 (PMC3237445; doi:10.1371/journal.pone.0028496)
Supplement: Table S10 — Breed, number (No.) of individuals, putative region of breed origina, breed class, and haplotype name of STR haplotypes included in NRY STR and SNP-STR analyses. Parentheses indicate individuals and haplotypes found in breed dogs that were genotyped in the present study and used in SNP-STR analysis; otherwise entries refer to published datab. (DOCX) [file pone.0028496.s012.docx]

Table S10. Breed, number (No.) of individuals, putative region of breed origin^a^, breed class, and haplotype name of STR haplotypes included in NRY STR and SNP-STR analyses. Parentheses indicate individuals and haplotypes found in breed dogs that were genotyped in the present study and used in SNP-STR analysis; otherwise entries refer to published data^b^.

| Breed | No. Individuals | Region | Breed Class | Haplotypes |
| --- | --- | --- | --- | --- |
| Afghan Hounds | 5 | SW Asia | Hound | 3i |
| Africanis | 6 | Africa | Sporting | 4h, 4i, 7a, 7f |
| Airedale Terrier | (2) | Europe | Sporting | (8a), (6p) |
| Akita | 15 | SE Asia | Working | 3a, 3b, 6k, 6q, 6r |
| American Cocker Spaniel | 26(2) | America | Sporting | 6b, 6f, (8d), (n1) |
| American Pit Bull Terrier | (2) | America | Non-Sporting | (8i), (9c) |
| Australian Shepherds | 20 | America | Herding | 7a, 7b, 7d, 7f |
| Basenji | 14 | Africa | Sporting | 4a, 4e, 4f, 4g |
| Basset Hounds | 12(1) | Europe | Hound | 6m, 6n, 6z, (6t) |
| Beagle | (3) | Europe | Sporting | (n9) |
| Bernese Mountain Dog | 11 | Europe | Working | 6k |
| Bichon Frise | 7(4) | Europe | Non-Sporting | (6p), 7d, (8d) |
| Border Collie | 17(1) | Europe | Herding | (6q), 7a, 7d, 7f |
| Borzoi | 10 | Europe | Hound | 7d |
| Boston Terrier | 15 | America | Working | 7a |
| Bouvier | 5 | Europe | Toy | 7e |
| Boxer | 26(2) | Europe | Working | 7c, (8i) |
| Brittany Spaniel | 5 | Europe | Sporting | 6ze |
| Brussels Griffon | (1) | Europe | Working | (8w) |
| Bulldog | 9(3) | Europe | Non-Sporting | 6p, 6zc, 6zd, (9c), (n10) |
| Bullmastiff | (1) | Europe | Working | (n10) |
| Canaan Dogs | 7 | SW Asia | Non-Sporting | 4j, 4k, 6e, 7c, 7e |
| Cavalier King Charles Spaniel | (1) | Europe | Toy | (6ze) |
| Chihuahua | 6(1) | America | Sporting | 7a, 6zg, 7d, (8d) |
| Chow Chow | 12 | SE Asia | Working | 3c, 7a |
| Dachshunds | 33 | Europe | Hound | 6i, 6q, 6s, 6t, 6u, 6v, 6za, 6zb, 7a, 7d |
| Breed | No. Individuals | Region | Breed Class | Haplotypes |
| Dalmation | 25 | Europe | Non-Sporting | 7b, 7e, 6n, 6t |
| Doberman Pinscher | 17(1) | Europe | Working | (n12), 6zg |
| English Springer Spaniel | 10 | Europe | Sporting | 6z, 7b |
| German Shepherd | 43(1) | Europe | Herding | 6e, 6h, 6o, 6s, 6zh, 6zi, 6zj, (8a) |
| Golden Retriever | 57(4) | Europe | Sporting | 6zh, (8c), (10l) |
| Greyhound | 15 | Europe | Hound | 7d, 7e |
| Jack Russell Terrier | 17(3) | Europe | Terrier | 6a, 6u, 6zc, 7a, 7d, (8d), (n7), (0f) |
| Keeshond | 14 | Europe | Non-Sporting | 6g, 6l, 6n |
| Labrador Retriever | 57(8) | America | Sporting | 6c, 6d, 6e, 6n,(6p), 6s, 6z, 6zh, 7a, 7d, (8c), (n3), (n5) |
| Long haired Dashund | (1) | Europe | Hound | (9c) |
| Maltese | 17(3) | Europe | Toy | 6j, (6p), 6t, 6w, 7d, (n8) |
| Mastiff | 5 | Europe | Working | 6y, 6zc |
| Miniature Pinscher | (1) | Europe | Toy | (n12) |
| Miniature Schnauzer | 23(1) | Europe | Working | 6q, 6zf, 6zh, 7a, 7e, (10l) |
| Mixed Breeds | (28) |  |  | (6p), (8w), (n2), (n14), (8g), (8d), (0f), (n10), (n6), (n8), (n11), (10l), (n5), (n13), (6q), (n4), (9a), (3d), (8a), (8b), (9c) |
| Newfoundland | 10 | America | Working | 6q, 6zh, 6zi |
| Norwegian Elkhound | 12 | Europe | Hound | 6x, 7e |
| Pembroke Welsh Corgi | 7 | Europe | Herding | 7d |
| Pomeranian | 14(3) | Europe | Toy | 6zg, 7a, (n12) |
| Poodle | 16(1) | Europe | Non-Sporting | 6q, (6p), 7a, 7d, 7e |
| Poodle (Miniature) | (1) | Europe | Non-Sporting | (n12) |
| Breed | No. Individuals | Region | Breed Class | Haplotypes |
| Poodle (Toy) | (2) | Europe | Toy | (6p), (8d) |
| Pugs | 23(2) | SE Asia | Toy | 3h, 6u, 7a, 7e, (8d) |
| Rhodesian Ridgeback | 12 | Africa | Hound | 4c, 4d |
| Rottweiler | 38 | Europe | Working | 6q, 6r |
| Saint Bernard | (1) | Europe | Working | (n12) |
| Saluki | 10(31) | SW Asia | Hound | 4b, 7c, (10a), (8h), (8i), (8j), (8n), (9g), (9o), (9p) |
| Scottish Terrier | 14 | Europe | Terrier | 7e |
| Sharpei | 11 | SE Asia | Non-Sporting | 3e, 3f, 6s, 7c |
| Shetland Sheepdog | 21(3) | Europe | Herding | 6k, 6q, 7e, (8x) |
| Shih Tzu | 23(1) | SE Asia | Non-Sporting | 3h, 7d, 7e, (6p) |
| Silky Terrier | (1) | Australia | Toy | (6p) |
| Tibetan Terrier | 5 | SE Asia | Terrier | 3d, 3g |
| Weimeraner | 5 | Europe | Sporting | 7e |
| West Highland White Terrier | 16(1) | Europe | Terrier | 7d, 7e, (8w) |
| Yorkshire Terrier | 20(3) | Europe | Toy | (6p), 7d |
| Total | 913 |  |  |  |

^a^Fogel B (1995) The Encyclopedia of the Dog. New York: DK Publishing.416 p.

^b^Bannasch D, Bannasch M, Ryun J, Famula T, Pedersen N (2005) Y chromosome haplotype analysis in purebred dogs. Mamm Genome 16: 273-280.
